# Standardized Short-Wave Infrared Hyperspectral Imaging for Non-Destructive Spectral Comparison of Coin Denominations, Design Variants, and Surface-Use Conditions

**DOI:** 10.3390/s26196264

**Published:** 2026-10-02

**Authors:** Hsin-Che Wang, Kuo-Chih Su, Yi-Jing Sheen, Chiu-Chin Sung, Min-Yi Chou, Kuan-Jung Pan, Hsian-Min Chen

**Affiliations:** 1 Center for Quantitative Imaging in Medicine (CQUIM), Department of Medical Research, Taichung Veterans General Hospital, Taichung 407219, Taiwan; leowanghsi@vghtc.gov.tw (H.-C.W.); docmedjean@vghtc.gov.tw (Y.-J.S.); chew40428@vghtc.gov.tw (C.-C.S.); febe901119@vghtc.gov.tw (M.-Y.C.); rong-pan26@vghtc.gov.tw (K.-J.P.); 2 Department of Medical Research, Taichung Veterans General Hospital, Taichung 407219, Taiwan; kcsu@vghtc.gov.tw; 3 Department of Medical Equipment Development and Application, Hung Kuang University, Taichung 433304, Taiwan; 4 Division of Endocrinology and Metabolism, Department of Internal Medicine, Taichung Veterans General Hospital, Taichung 407219, Taiwan; 5 Department of Medicine, School of Medicine, National Yang Ming Chiao Tung University, Taipei 112304, Taiwan; 6 Department of Post-Baccalaureate Medicine, National Chung Hsing University, Taichung 402202, Taiwan; 7 Department of Biomedical Engineering, China Medical University, Taichung 404333, Taiwan

**Keywords:** short-wave infrared hyperspectral imaging, coin analysis, non-destructive testing, spectral angle mapper, acquisition standardization, spectral comparison

## Abstract

**Highlights:**

**What are the main findings?**

- A standardized 900–1700 nm SWIR-HSI workflow was developed for non-destructive spectral analysis of metallic coins.
- Under controlled imaging conditions, spectral differences among new and circulated coins, NT$10 design variants, and different denominations were quantified using SAM.

**What are the implications of the main findings?**

- Imaging standardization is essential for reliable SWIR-HSI analysis of highly reflective metallic coins.
- The proposed workflow provides a methodological basis for further validation of controlled and non-destructive spectral analysis of metallic coins.

**Abstract:**

Coins contain multiple types of information, including denomination, design variant, year of issue, and surface condition. This study established a standardized 900–1700 nm short-wave infrared hyperspectral imaging (SWIR-HSI) workflow for controlled and non-destructive spectral comparison of metallic coins. Twenty-five independent New Taiwan dollar (NT$10) coins were used to evaluate imaging position, coin side, and aperture setting. At position 13 on the reverse side, the mean spectral standard deviations were 0.192, 0.142, and 0.151 for F1.8, F2.8, and F4, respectively, and F2.8 was selected for subsequent measurements. Using the same NT$10 coin, support-material and in-plane rotation experiments produced spectral angle mapper (SAM) ranges of 0.232–1.087 and 0.132–0.581, respectively. Under the standardized conditions, the SAM value between one new and one circulated NT$10 coin was 0.729. Pairwise SAM values ranged from 0.260 to 1.680 among eight NT$10 design variants and from 0.458 to 2.449 among six denomination or special-year samples. An initial repeatability evaluation under the current experimental conditions, using three additional NT$10 coins and 24 acquisitions, yielded mean SAM values of 0.2450°, 0.6368°, and 0.3323° for repositioning, inter-day, and inter-operator comparisons, respectively, with an overall median spectral CV of 0.83%. These results quantitatively demonstrate that acquisition conditions affect SWIR spectral measurement and that selected coin samples exhibit measurable spectral differences under controlled conditions. The present study focuses on acquisition standardization and descriptive spectral comparison and does not establish validated coin classification, material identification, or surface-condition assessment.

## 1. Introduction

Coins contain many types of information, including denomination, design, year, material composition, and surface preservation condition. Therefore, non-destructive analysis of coins has potential value in coin identification, collection preservation, numismatic studies, material characterization, and educational display. Traditional coin identification often depends on visual inspection, weight and size measurement, or common visible-light images to recognize patterns and shape features. Previous review studies on ancient and modern coin recognition have reported that automatic coin recognition systems can be mainly divided into mechanical, electromagnetic, and image-processing methods. Among these methods, image-processing approaches mainly focus on the appearance, contour, and pattern features of coins [1]. However, during long-term circulation and storage, coins may be affected by wear, oxidation, dirt, changes in surface roughness, and different lighting conditions. As a result, methods that rely only on visual images or human judgment may lack enough objectivity and repeatability. Therefore, developing a non-contact, non-destructive, and quantitative method for analyzing coin surface features is valuable for research and practical applications.

Previous studies on coin identification have mainly focused on visible-light images and computer vision methods. For example, coins can be classified based on patterns, text, relief structures, edge contours, or deep learning models. Anwar et al. [2] used feature fusion and an attention mechanism to classify ancient Roman Republican coins. They mainly used the reverse-side patterns of coins as the recognition target, and their results showed that deep learning can perform well in ancient coin image classification. However, these methods mainly depend on the shape and image features of coins under visible light. They are useful for denomination recognition, ancient coin pattern classification, and automatic image retrieval. However, they are less able to provide information related to surface condition, oxidation level, contaminants, material differences, or local spectral reflectance features. On the other hand, analytical techniques such as X-ray fluorescence (XRF), Raman spectroscopy, and scanning electron microscopy–energy dispersive X-ray spectroscopy (SEM-EDS) can provide more direct information about elemental or chemical composition. These methods have also been used to analyze the materials and corrosion products of ancient coins [3,4]. However, they are mostly point-based or local analysis methods. Their equipment cost, operating requirements, and measurement efficiency may limit their use as fast tools for whole-surface screening.

Hyperspectral imaging (HSI) combines the spatial information of conventional images with the spectral information of spectroscopy. It can obtain a reflectance spectrum at each pixel over continuous wavelengths. Therefore, it can detect subtle spectral differences that are difficult to observe using common red–green–blue (RGB) images. Compared with point-based spectrometers, HSI can preserve both the spatial distribution and spectral variation of a sample surface. Therefore, it is suitable for non-contact and non-destructive surface analysis. Liang reported that multispectral and hyperspectral imaging can rapidly scan large objects and provide both spatial and spectral information. For this reason, these methods have been widely used in art conservation, art history, and archaeological research [5]. In addition, Picollo et al. [6] reported that HSI systems in the cultural heritage field are often operated in reflectance mode. These systems can cover visible (Vis), near-infrared (NIR), and short-wave infrared (SWIR) wavelength ranges. They can reconstruct reflectance spectra at each pixel and provide information close to laboratory-based spectral analysis. In recent years, HSI has also been applied to cultural heritage preservation, artwork inspection, document and banknote analysis, material classification, and surface defect detection. These applications show that HSI has good potential for non-destructive analysis of materials and surface features [5,6,7,8]. Recent studies also show that HSI is being used in a wider range of sensing applications, including food quality inspection, plant phenotyping, spectral camera development, and industrial or biological analysis [9,10,11,12,13]. These studies demonstrate the growing value of HSI as a non-contact and non-destructive sensing tool. At the same time, recent work has also emphasized that HSI data quality depends not only on algorithms or classification models, but also on system performance, calibration, metadata, and image acquisition workflow [9,10,12]. However, in many application studies, less attention is given to how practical acquisition factors, such as sample position, illumination geometry, aperture setting, background material, and object orientation, affect spectral repeatability before spectral comparison. This issue is especially important for metallic coins because their high reflectance and relief structures make their spectra sensitive to acquisition conditions. Therefore, acquisition standardization should be considered an essential step before using SWIR-HSI for reliable coin spectral comparison.

Among the different hyperspectral wavelength ranges, the short-wave infrared (SWIR) range is sensitive to variations in surface reflectance and may show spectral responses associated with corrosion products, contaminants, roughness, and material absorption features. However, such spectral responses are not specific to a single physical or chemical factor. Therefore, in the present study, 900–1700 nm SWIR-HSI was used to compare overall spectral differences among selected coin samples rather than to identify alloy composition, oxidation level, or surface-preservation state. De Kerf et al. [14,15] used 900–1700 nm SWIR-HSI to identify corrosion minerals on carbon steel surfaces. They further used X-ray diffraction (XRD) as reference data and combined it with SWIR-HSI for quantitative detection of corrosion minerals. These studies showed that SWIR-HSI has potential for analyzing corrosion products and surface condition changes on metal surfaces. However, metallic coins have high reflectance and surface relief structures. Their SWIR reflectance signals can be easily affected by specular reflection, illumination angle, lens aperture, imaging position, background support material, and coin rotation direction. Among these factors, illumination configuration is particularly critical for metallic coins because directional and specular reflections from the reflective surface and relief structures can substantially alter the measured reflectance signal. Therefore, illumination geometry should be treated as a primary experimental control in SWIR-HSI acquisition of metallic coins. If standardized imaging conditions are not established first, the observed spectral differences may include variations caused by the image acquisition conditions. This may reduce the reliability and repeatability of the analysis results.

Although HSI has been applied to studies on cultural heritage, banknotes, anti-counterfeiting materials, and metal surface corrosion, SWIR-HSI analysis of metallic coins is still relatively limited. Lim and Murukeshan [7] used push-broom HSI to build a spectral library of polymer banknotes. Their study showed that HSI can be used for spectral recording and analysis of currency objects. Huang et al. also reported in a review of anti-counterfeiting applications that HSI has been used for the detection of documents, artworks, photographs, holograms, and currency [8]. However, most previous studies focused on banknotes, documents, or anti-counterfeiting materials, and relatively few studies have systematically investigated SWIR-HSI analysis of metallic coins. Three related but distinct issues should be considered in this context. First, acquisition standardization concerns how imaging factors such as position, aperture, support material, illumination geometry, and coin orientation affect spectral measurement stability. Second, spectral interpretation concerns whether measured differences can be associated with coin characteristics such as surface-use condition, design variant, or denomination; such differences should not be interpreted as direct evidence of material composition, oxidation level, or surface-preservation state without independent reference measurements. Third, automatic classification requires sufficiently large independent datasets, appropriate data splitting, and validated classification performance. The present study focuses on the first issue and on descriptive spectral comparison under standardized conditions, but does not attempt material identification or automatic coin classification.

Based on the above background, this study was designed as a feasibility and acquisition-standardization investigation of 900–1700 nm SWIR-HSI for non-destructive spectral analysis of metallic coins. We tested two methodological hypotheses: (1) practical acquisition factors, including imaging position, coin side, aperture setting, support material, and coin rotation, produce measurable variation in SWIR spectral measurements; (2) after these acquisition factors are controlled, selected coin samples with different surface-use conditions, design variants, or denominations retain quantifiable spectral differences at the coin level.

**Figure 1:**
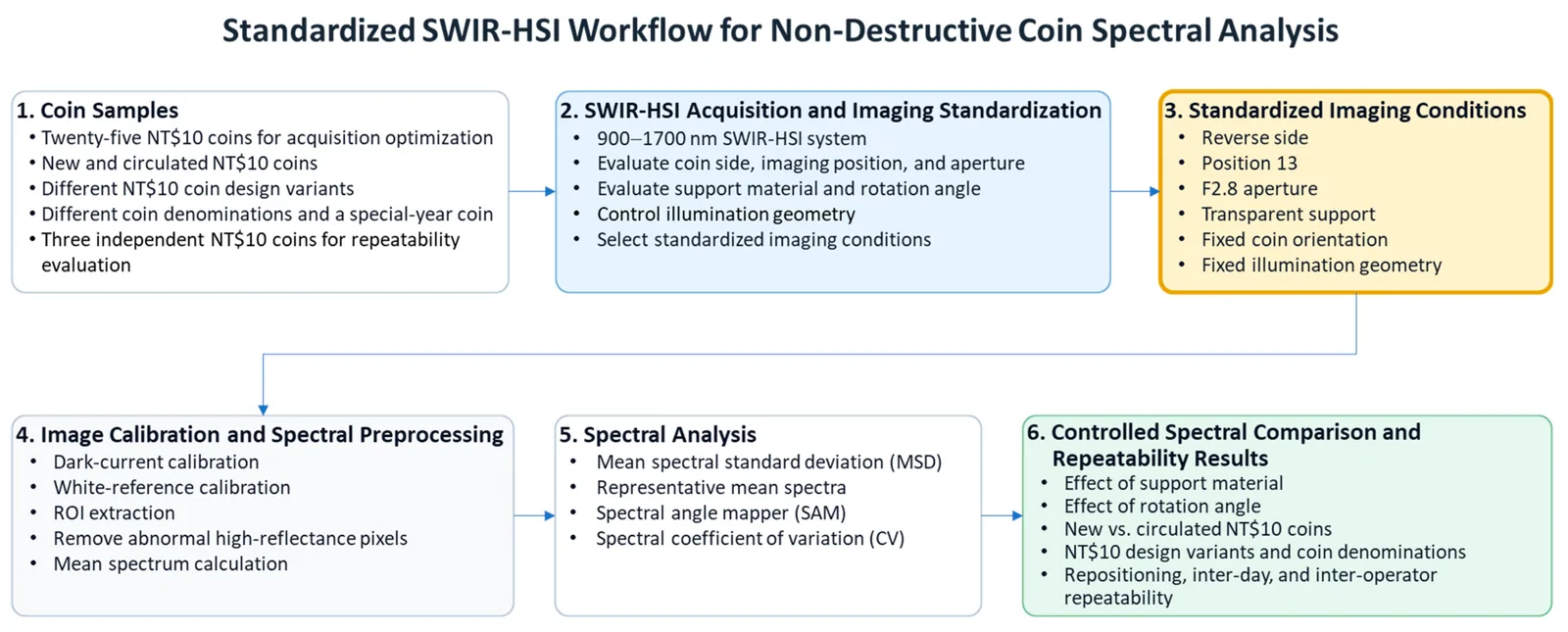
Overall workflow of the proposed standardized short-wave infrared hyperspectral imaging (SWIR-HSI) method for non-destructive spectral analysis of coins.

Accordingly, the objectives of this study were: (1) to quantify the effects of these acquisition factors on spectral measurement; (2) to establish controlled imaging conditions for subsequent spectral comparison; and (3) to descriptively compare the SWIR spectra of selected coin samples under the standardized acquisition conditions. The study was not designed to determine alloy composition, oxidation level, or surface-preservation state, and it did not develop or validate an automatic coin classification system. The overall workflow of the proposed standardized SWIR-HSI method is summarized in Figure 1.

## 2. Materials and Methods

### 2.1. Coin Samples and Support Materials

This study used circulating coins from Taiwan and a special-year coin as samples for short-wave infrared hyperspectral imaging. The coin samples included New Taiwan dollar (NT$) 1, NT$5, NT$10, NT$20, and NT$50 coins. An NT$0.5 coin from Republic of China (ROC) Year 38 was also included as a special-year coin sample. For NT$10 coins, this study further included new coins, circulated coins, and different NT$10-coin design variants. These samples were used to compare short-wave infrared spectral differences associated with different surface-use conditions, design variants, and coin denominations under controlled imaging conditions. The coin samples, support conditions, imaging positions, aperture settings, and main purposes of each experiment are summarized in Table 1.

The number and characteristics of the independent coin samples used in each experiment were documented separately. For the imaging-position and aperture optimization experiment, 25 independent NT$10 coins were used. All 25 coins were issued in ROC Year 112 and were new, uncirculated coins randomly selected from a batch of 200 new NT$10 coins obtained from the Bank of Taiwan. For the support-material and rotation-angle experiments, one NT$10 coin was randomly selected from the same batch of new, uncirculated coins for each experiment.

For the comparison between new and circulated NT$10 coins, one new NT$10 coin was randomly selected from the same batch of 200 new coins, and one circulated NT$10 coin issued in ROC Year 111 was randomly selected from coins available in our laboratory. The design-variant experiment included eight independent NT$10 coins, with one coin representing each design variant. The denomination experiment included six independent coins: NT$50, NT$20, NT$10, NT$5, NT$1, and one NT$0.5 coin issued in ROC Year 38. These samples were used as representative coins for controlled spectral comparison rather than for population-level statistical inference. Photographs of the actual coin samples used in these experiments are provided in Supplementary Figure S1, and detailed sample information is summarized in Supplementary Table S1.

All coins were kept in their original surface conditions before imaging. No chemical cleaning, polishing, or other surface treatment was performed. This was done to avoid changing surface features such as oxidation, wear, contaminants, and roughness. If visible dust was present on the coin surface, it was gently removed using a dry lint-free cloth. This step was used to reduce the effect of loose surface dust on the reflectance signal. Since this study mainly analyzed relative spectral differences among different coin types and imaging conditions, all samples were analyzed using the same hyperspectral imaging system, the same image calibration procedure, and standardized imaging conditions.

**Figure 2:**
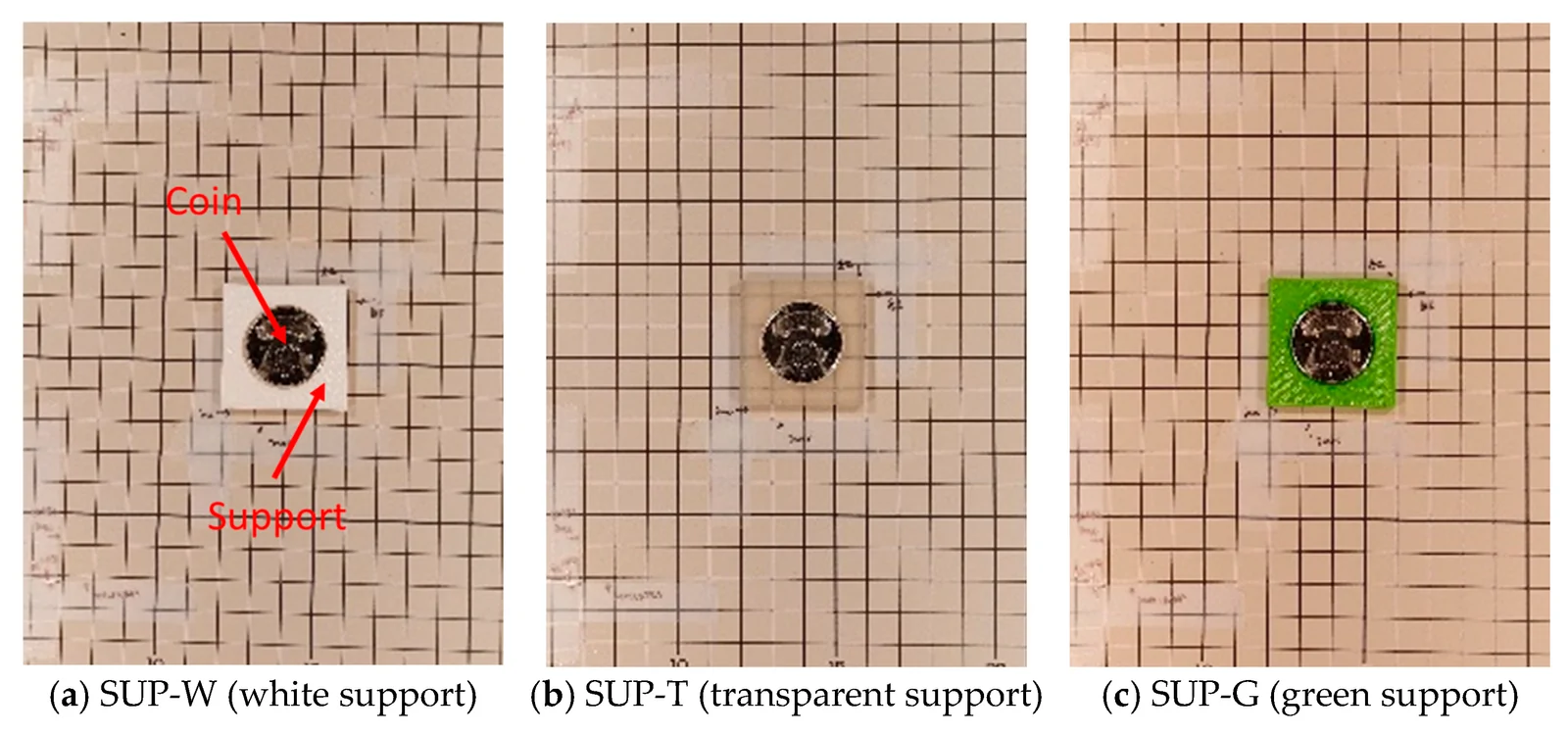
Actual products of the three 3D-printed coin supports used in this study: (**a**) white support (SUP-W), (**b**) transparent support (SUP-T), and (**c**) green support (SUP-G). A representative coin placed on each support is shown. The coin and support are indicated in panel (**a**) for illustration.

In addition to coin samples, this study used three-dimensional (3D) printing to make three different supports. These included a green support, a transparent support, and a white support, which were labeled as SUP-G, SUP-T, and SUP-W, respectively. The actual products of the three supports are shown in Figure 2. The color, material, fabrication method, purpose, and later use of each support are summarized in Table 2. The supports were designed to fix the coin position and keep the imaging surface of coins with different thicknesses at the same height. This design was used to reduce changes in working distance, focus, and illumination geometry caused by coin thickness differences. Since coins with different denominations may have different thicknesses, placing them directly on the same flat surface may lead to different top-surface heights. This could affect short-wave infrared reflectance measurement. Therefore, the supports were designed to control the coin imaging height and improve the repeatability of spectral comparison among different coins.

This study first compared the short-wave infrared spectra of the same NT$10 coin placed on the three supports. This experiment was used to evaluate whether support material could affect spectral measurement of coins. The results of the support comparison were used to confirm whether support material was a control factor that could influence the spectra. In the following single-coin experiments, including coin rotation, new and circulated coin comparison, different NT$10-coin design variants, and different coin denominations, all coins were imaged using the transparent support. This kept the support condition consistent across all comparisons and prevented support material from becoming a source of variation between groups.

### 2.2. Short-Wave Infrared Hyperspectral Imaging System and Imaging Setup

**Figure 3:**
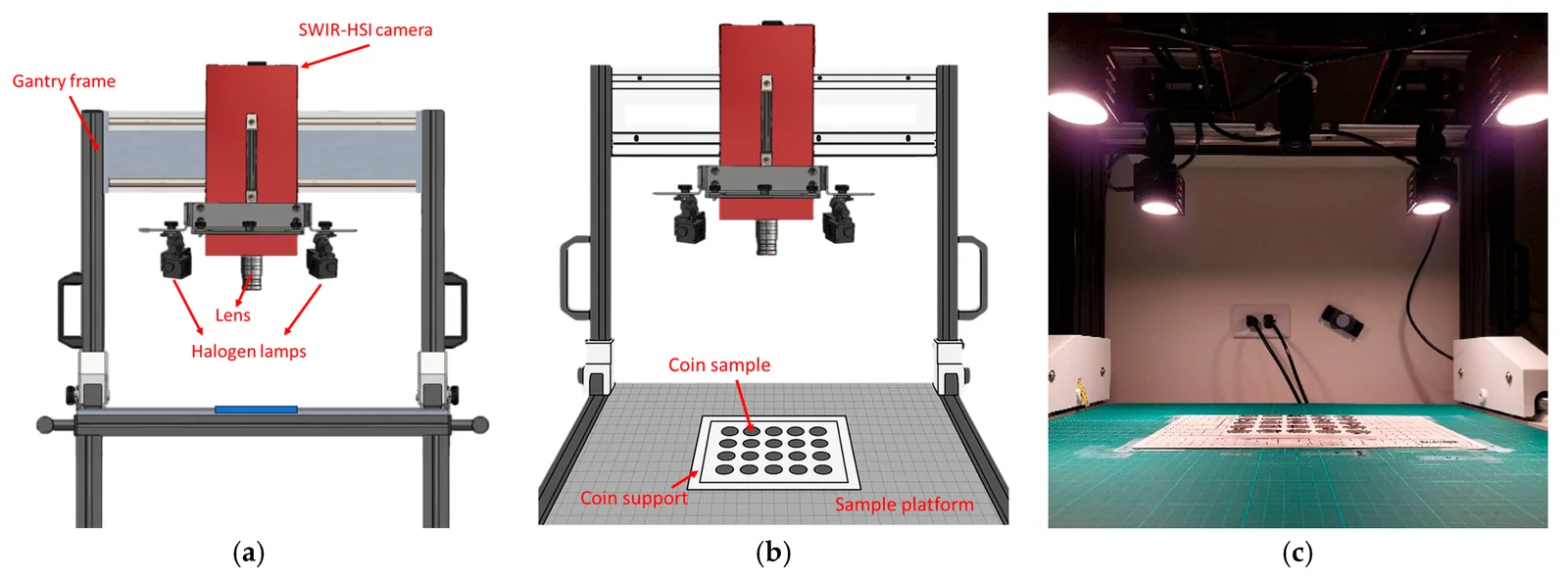
Schematic diagram and actual setup of the SWIR hyperspectral imaging system for coin imaging. (**a**) Front-view schematic showing the SWIR-HSI camera, lens, halogen illumination sources, and gantry frame; (**b**) perspective-view schematic showing the sample platform, coin support, and coin imaging position; (**c**) photograph of the actual imaging setup.

This study used a 900–1700 nm short-wave infrared hyperspectral imaging system to acquire reflectance spectral images of coins. The system mainly included a short-wave infrared hyperspectral camera, a lens, a fixed gantry frame, illumination sources, and a sample platform. The hyperspectral camera was fixed vertically at the center of the gantry frame, with the lens facing downward toward the imaging platform. The illumination sources were placed on both sides of the camera to provide stable and symmetric lighting for the coin imaging area. The mechanical design of the hyperspectral imaging system and the actual imaging setup are shown in Figure 3. The hyperspectral imaging system and main imaging parameters used in this study are summarized in Table 3.

The imaging setup used an aluminum extrusion gantry frame as the main support structure to maintain a stable relative position among the camera, light sources, and sample platform. The coin sample was placed in the support on the imaging platform and positioned at the selected location within the camera field of view. The optical axes of the four halogen lamps were directed vertically downward, approximately parallel to the hyperspectral camera optical axis and normal to the sample plane. No inward tilt toward the coin was applied because directing the illumination toward the highly reflective metallic surface produced strong reflected signals. During image acquisition, the camera height, lens focus, light-source position, illumination geometry, and sample-platform position were kept fixed. The camera–sample distance was maintained at 30.5 cm, and the exposure time was fixed at 55 ms throughout these six primary experiments.

The six primary experiments described in Section 2.3.1, Section 2.3.2, Section 2.3.3, Section 2.3.4, Section 2.3.5 and Section 2.3.6 were completed within the same day using the same SWIR-HSI system and fixed imaging configuration. After system startup, the four halogen lamps were allowed to warm up for approximately 13–15 min. An initial white-reference image was then acquired, and formal image acquisition was started when the maximum digital number (DN) measured from the white reference reached approximately 11,800 ± 100 DN. During the experiments, a new white-reference image was acquired after every four hyperspectral image acquisitions, and the most recently acquired white-reference image was used for subsequent relative reflectance calibration. This procedure was used to reduce the influence of temporal variation in illumination output and detector response. Lamp-output stability was not independently quantified using a separate radiometric stability test.

At the end of the same-day acquisition session, immediately before system shutdown, one dark-current hyperspectral image was acquired by completely covering the camera lens with the lens cap to block incident light. This image was used as the dark-current reference for all hyperspectral images acquired during that session. Repeated dark-current measurements were not performed; therefore, temporal variation in the detector dark signal was not independently quantified.

For white-reference calibration, a WC-430 99% Spectralon calibrated white plate (purchased through ISUZU OPTICS Co., Ltd., Hsinchu, Taiwan), designed for the 250–2500 nm spectral range, was used. The white-reference surface was positioned at the same imaging height as the coin surface and was imaged under the same fixed camera and illumination geometry.

The raw hyperspectral images were calibrated using the white-reference image and the dark-current image, as shown in Equation (1):

(1) $$R\left(\lambda \right)=\frac{I_{\mathrm{raw}}\left(\lambda \right)-I_{\mathrm{dark}}\left(\lambda \right)}{I_{\mathrm{white}}\left(\lambda \right)-I_{\mathrm{dark}}\left(\lambda \right)}$$

where *R*(*λ*) is the relative reflectance at wavelength *λ*, *I*_raw_(*λ*) is the raw image signal of the coin, *I*_dark_(*λ*) is the dark-current image signal, and *I*_white_(*λ*) is the corresponding WC-430 white-reference signal. All calibrated images were subsequently used for ROI extraction and spectral analysis.

To establish the standardized imaging workflow, a preliminary experiment was performed to evaluate the effects of different imaging positions and aperture settings on spectral variation, as described in Section 2.3.1.

Based on this optimization process, all subsequent spectral comparison experiments were performed at position 13 using an F2.8 aperture setting. To reduce differences in imaging height caused by different coin thicknesses, all coins were placed in a standardized transparent support. This kept the imaging surface of the coins at the same height. This design reduced the effects of differences in working distance, focus, and illumination geometry on the reflectance spectra.

### 2.3. Experimental Design for Coin Hyperspectral Imaging

#### 2.3.1. Optimization of Imaging Position and Aperture Conditions

**Figure 4:**
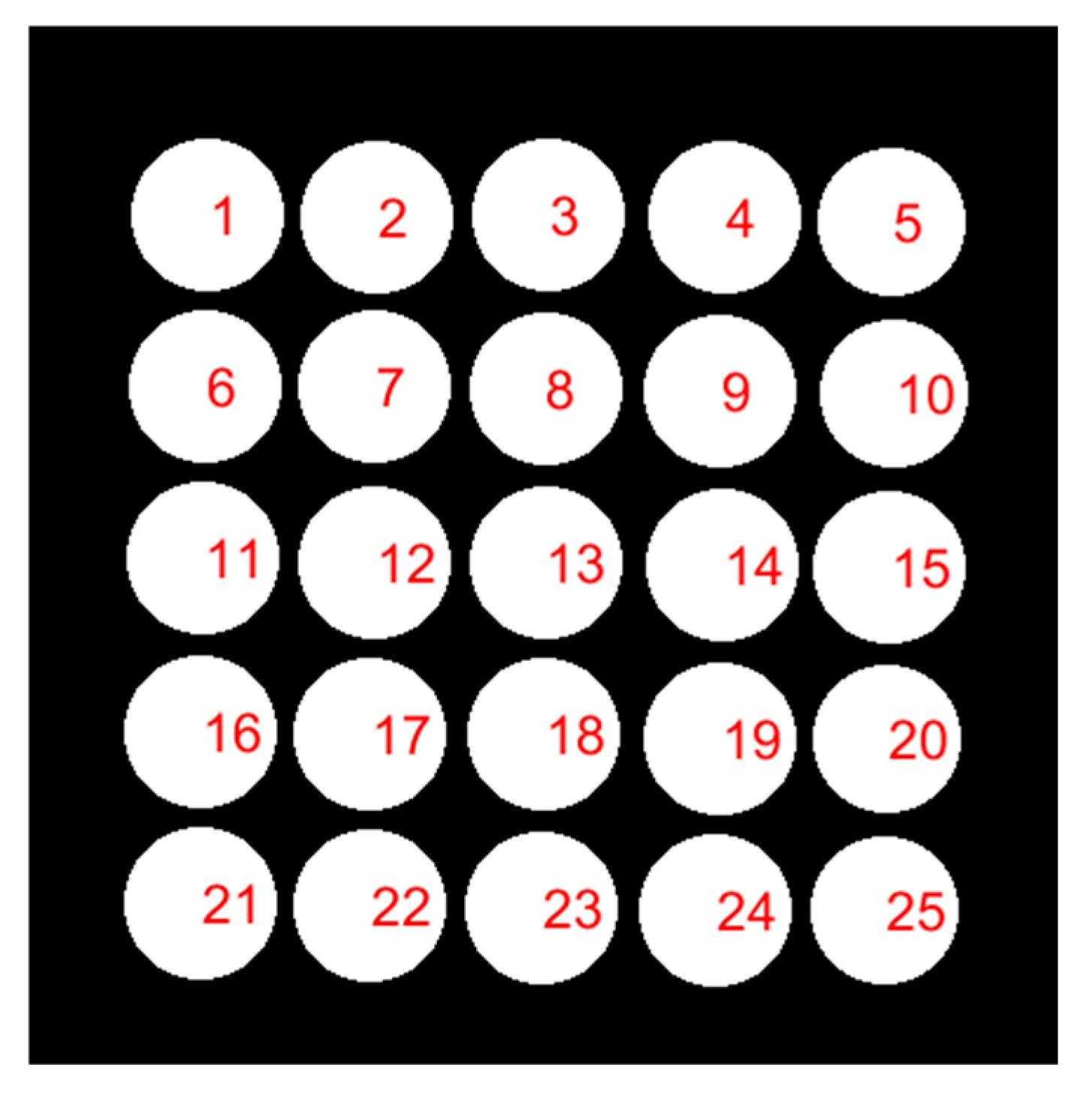
Schematic diagram of the 5 × 5 numbering layout for coin imaging positions. In this study, 25 NT$10 coins were placed on a white support at the same time and arranged in a 5 × 5 layout, corresponding to 25 imaging positions. The positions were numbered from left to right and from top to bottom. This layout was used to evaluate spectral stability under different imaging positions, coin sides, and aperture conditions.

To establish stable and repeatable imaging conditions for coin short-wave infrared hyperspectral imaging (SWIR-HSI), this study first evaluated the effects of imaging position, coin side, and lens aperture setting on the spectral variation of NT$10 coins. To avoid changes in light intensity, exposure batch, or environmental conditions caused by moving and imaging the coins one by one, 25 NT$10 coins were placed on the same white support at the same time. The coins were arranged in a 5 × 5 layout, corresponding to 25 imaging positions. The positions were numbered from position 1 to position 25 from left to right and from top to bottom, as shown in Figure 4.

In this experiment, the obverse and reverse sides of the 25 NT$10 coins were imaged separately. The obverse side was defined as the portrait side and was labeled as H. The reverse side was defined as the side with text and denomination marks and was labeled as W. To evaluate the effect of aperture setting on spectral stability, images were acquired using three lens aperture settings: F1.8, F2.8, and F4. Therefore, this experiment included six imaging conditions: F1.8_H, F2.8_H, F4_H, F1.8_W, F2.8_W, and F4_W.

Under each imaging condition, the 25 coins were acquired in the same SWIR-HSI image. The images were converted into relative reflectance images using the same white-reference and dark-current calibration procedure. After calibration, each coin area was first segmented to obtain the region of interest (ROI) for each position. Next, the reflectance values of all pixels in each ROI were extracted at each spectral band. The standard deviation of pixel reflectance within the ROI was then calculated for each band. Finally, the standard deviations across all bands were averaged and used as the mean spectral standard deviation for that position under that imaging condition.

The mean spectral standard deviation was calculated as shown in Equation (2):

(2) $${\mathrm{MSD}}_{p,a,s}=\frac{1}{N_{\lambda }}\sum_{\lambda =1}^{N_{\lambda }}\mathrm{SD}\left(R_{p,a,s,\lambda }\right)$$

where *MSD_p_*_,*a*,*s*_ is the mean spectral standard deviation at imaging position *p*, aperture setting *a*, and coin side *s*. *N_λ_* is the number of spectral bands. *R_p_*_,*a*,*s*,*λ*_ is the pixel reflectance value of the coin ROI at wavelength *λ* under the corresponding position, aperture, and coin side. *SD* represents the standard deviation. A lower mean spectral standard deviation indicates smaller spectral variation among pixels within the ROI. This means better image measurement stability.

It should be noted that this experiment was performed by imaging 25 NT$10 coins at the same time. Therefore, the mean spectral standard deviation at each position reflects the overall spectral stability of the coin ROI at different positions within the same imaging batch. This design was mainly used to select a more stable and consistent imaging position, coin side, and aperture condition for later coin imaging. It was not intended to evaluate the absolute position effect of the same coin placed at different positions.

#### 2.3.2. Effect of Support Material on the Spectra of NT$10 Coins

To evaluate whether the surrounding support affected SWIR spectral measurement, the same NT$10 coin was sequentially imaged on three 3D-printed supports: green (SUP-G), transparent (SUP-T), and white (SUP-W). The color, material, fabrication method, and purpose of each support are summarized in Table 2. Because metallic coins are highly reflective and contain edges and relief structures, the surrounding support may indirectly influence the measured reflectance through reflection, scattering, or edge-related optical effects.

All acquisition parameters other than support material were kept fixed. The acquired images were analyzed using the common calibration and spectral-analysis procedures described in Section 2.2 and Section 2.4. Based on this comparison, SUP-T was selected as the standard support for all subsequent single-coin experiments to maintain a consistent support condition.

#### 2.3.3. Effect of Coin Rotation Angle on Spectral Measurement

After the imaging position, aperture, and support condition were standardized, the effect of in-plane coin rotation was evaluated using the same NT$10 coin at 0°, 90°, 180°, and 270°. The corresponding physical orientations of the coin are illustrated in Supplementary Figure S1c. Coin orientation was examined because rotation may change the relative relationship among surface relief, incident illumination, and camera viewing direction, thereby affecting the measured reflectance. All other acquisition conditions were kept fixed, and the images were analyzed using the procedures described in Section 2.2 and Section 2.4. The purpose of this experiment was to determine whether coin orientation should be controlled in the standardized acquisition workflow.

#### 2.3.4. Comparison Between New and Circulated NT$10 Coins

After evaluating the image acquisition conditions, including imaging position, aperture setting, support material, and coin rotation angle, this study further applied the standardized imaging workflow to conduct a preliminary spectral comparison between a new and a circulated NT$10 coin. During circulation and storage, coins may undergo wear, oxidation, contamination, changes in surface roughness, or changes in local reflectance properties. These factors may affect their short-wave infrared reflectance spectra. Therefore, this experiment compared new and circulated NT$10 coins to examine whether coins with different surface-use conditions showed measurable spectral differences under the standardized imaging conditions.

One new NT$10 coin and one circulated NT$10 coin were used for this preliminary spectral comparison. The new coin was issued in ROC Year 112 and was randomly selected from the same batch of 200 new, uncirculated NT$10 coins obtained from the Bank of Taiwan. The circulated coin was issued in ROC Year 111 and was randomly selected from coins available in our laboratory. The circulated coin had been used in daily circulation and showed visible signs of surface use. No chemical cleaning, polishing, or other surface treatment was performed before imaging in order to preserve the original surface condition of each coin. The two coins represented different surface-use conditions but were not intended to represent the full variability of new and circulated NT$10 coins.

Both coins were imaged under the standardized acquisition conditions and analyzed using the common calibration and spectral-analysis procedures described in Section 2.2 and Section 2.4. This comparison was used to determine whether the selected new and circulated coins exhibited quantifiable short-wave infrared spectral differences under the controlled imaging conditions. In this study, the term “surface-use condition” refers only to the documented sample status (new and uncirculated versus circulated) and is not intended as a quantitatively validated measure of wear, oxidation, or surface-preservation state. This experiment was intended as a preliminary spectral comparison between selected new and circulated coins and was not designed to establish a validated method for surface-condition assessment.

#### 2.3.5. Comparison Among Different NT$10-Coin Design Variants

Different NT$10-coin design variants differed in characteristics such as pattern, text layout, relief structure, year of issue, and observed surface-use history. These characteristics were not independently characterized in terms of material composition, oxidation level, or surface-preservation state. Therefore, the purpose of this experiment was limited to comparing the overall spectral features of the selected design-variant samples under the same controlled imaging conditions.

Eight independent NT$10 coins were included in this experiment, with one coin representing each design variant. Therefore, the purpose of this experiment was to compare the spectra of the selected representative design variants under controlled imaging conditions, rather than to characterize within-variant population variability.

All samples were imaged under the standardized acquisition conditions and analyzed using the common calibration and spectral-analysis procedures described in Section 2.2 and Section 2.4. The resulting representative mean spectra were descriptively compared among the selected design variants.

#### 2.3.6. Comparison Among Different Coin Denominations and a Special-Year Coin

Six independent coins were included in the denomination comparison, with one coin representing each denomination or special-year category: NT$50, NT$20, NT$10, NT$5, NT$1, and ROC Year 38 NT$0.5. Therefore, this experiment was intended as a controlled comparison of the selected representative coin samples rather than a population-level comparison among denominations.

All samples were imaged under the standardized acquisition conditions described above. The transparent support was used to maintain a consistent imaging-surface height across coins with different thicknesses. The acquired images were analyzed using the common calibration and spectral-analysis procedures described in Section 2.2 and Section 2.4.

The mean spectra of different coin denominations and the special-year coin were then analyzed using spectral curves and spectral angle differences. These analyses were used to descriptively quantify spectral similarities and differences among the selected coin samples rather than to evaluate statistical class separability.

Because no independent elemental-composition analysis was performed, the observed spectral differences were not interpreted as evidence of specific alloy composition.

#### 2.3.7. Initial Repeatability Evaluation of the Standardized Acquisition Workflow

To provide an initial quantitative evaluation of repeatability under the current experimental conditions, three independent new and uncirculated NT$10 coins were randomly selected from the same batch of new ROC Year 112 NT$10 coins described in Section 2.1. Each coin was imaged on two separate measurement days by two operators. For each operator on each measurement day, each coin was imaged twice. After the first acquisition, the coin was completely removed from the transparent support and then repositioned before the second acquisition. Thus, the repeatability experiment consisted of 3 coins × 2 measurement days × 2 operators × 2 repositioned acquisitions, resulting in a total of 24 hyperspectral acquisitions.

All acquisitions were performed using the standardized conditions established in this study: the reverse side of the coin, position 13, F2.8 aperture, transparent support, fixed coin orientation, fixed illumination configuration, a camera–sample distance of 30.5 cm, and an exposure time of 55 ms. On each measurement day, the SWIR-HSI system was independently started and the halogen lamps were warmed up for approximately 13–15 min. White-reference and dark-current calibration were performed according to the procedure described in Section 2.2. A new white-reference image was acquired after every four hyperspectral acquisitions.

Each acquisition was processed independently using the ROI extraction, spectral preprocessing, abnormal high-reflectance pixel removal, and spectral aggregation procedures described in Section 2.4. The resulting representative coin-level mean spectra were used for repeatability analysis; individual ROI pixels were not treated as independent observations.

Repeatability was evaluated for three sources of measurement variation. Repositioning repeatability was assessed by comparing the two acquisitions of the same coin obtained by the same operator on the same day. Inter-day repeatability was assessed by comparing representative spectra of the same coin and operator between the two measurement days. Inter-operator repeatability was assessed by comparing representative spectra obtained from the same coin by the two operators under the same measurement-day condition. Spectral-shape repeatability was quantified using pairwise spectral angle differences calculated as described in Section 2.4.3, whereas reflectance-magnitude repeatability was evaluated using the spectral coefficient of variation (CV) across repeated acquisitions.

For each coin, eight representative coin-level mean spectra were obtained from the eight repeated acquisitions. At each of the 159 spectral bands, the CV was calculated from the eight corresponding mean reflectance values as the standard deviation divided by the mean reflectance and multiplied by 100 to express the value as a percentage. This resulted in 159 wavelength-specific CV values for each coin. The median of these 159 wavelength-specific CV values was used as the coin-level median spectral CV. The overall median spectral CV was then calculated as the median of the three coin-level median spectral CV values, and the reported range was defined by the minimum and maximum coin-level values. Thus, the spectral CV represents between-acquisition variability in the ROI-averaged coin-level mean reflectance across wavelengths rather than pixel-level variability within the ROI. Lower spectral angle differences and spectral CV values indicate greater measurement consistency.

### 2.4. Spectral Preprocessing and Spectral Difference Analysis

After dark-current and white-reference calibration, all coin hyperspectral images were converted into relative reflectance images. The images were then used for ROI extraction, mean spectrum calculation, and spectral difference analysis. Since the main purpose of this study was to compare short-wave infrared spectral differences under different imaging conditions, surface conditions, design variants, and coin denominations, all images were processed using the same calibration, preprocessing, and spectral analysis workflow. This ensured that the data from different groups were comparable.

#### 2.4.1. ROI Extraction and Mean Spectrum Calculation

In this study, Fiji ImageJ software (version 2.18.0) [16] was used to select the coin ROI. First, a band image in which the coin boundary could be clearly identified was selected from the hyperspectral image. Then, the circular selection tool in Fiji ImageJ was used to manually draw a circular ROI along the coin boundary. The selected ROI was converted into a binary mask. Pixels inside the mask were defined as the coin region, while pixels outside the mask were regarded as the background and were not included in the later spectral analysis.

Although the detector of the short-wave infrared hyperspectral imaging system used in this study covered a wavelength range of 900–1700 nm and contained 512 spectral bands, the actual wavelength range used for analysis was 901–1696 nm because of the sensor response efficiency. For each image, band images from the 14th to the 490th band were selected for analysis. These 477 band images were then averaged every three bands into one band image. Therefore, each final hyperspectral cube contained 159 spectral band images for subsequent data analysis.

Assume that there are *p* pixels within the coin ROI, and the hyperspectral vector of the *i*th pixel is denoted as *x_i_*. After arranging all hyperspectral vectors within the ROI, a hyperspectral vector matrix *X* can be formed as follows:

*X* = [*x*_1_,*x*_2_,…,*x_p_*] (3)

where the size of *X* is 159 × *p*. Each column of the matrix represents the complete hyperspectral vector of one pixel within the ROI and contains the reflectance values of that pixel at 159 spectral bands. Each row of the matrix represents the reflectance values of all pixels within the ROI at the same spectral band.

Because metallic coin surfaces exhibit strong directional and specular reflection, local surface regions may produce abnormally high calibrated relative reflectance values even under fixed acquisition conditions. After dark-current and white-reference calibration, a relative reflectance value greater than 1 indicates that the calibrated coin signal at that wavelength exceeded the corresponding white-reference-normalized level. For the highly reflective coin surfaces examined in this study, such values were considered primarily indicative of local specular-reflection artifacts or related geometric reflection effects, although minor calibration mismatch could also contribute. The reflectance > 1 criterion was therefore used as an operational artifact-rejection threshold rather than as a detector-saturation threshold. If these abnormal spectra were directly included in the mean-spectrum calculation, they could disproportionately increase the mean reflectance at affected wavelengths and reduce the stability of the representative coin spectrum.

Therefore, before calculating the mean spectrum, each hyperspectral vector within the ROI was screened for abnormally high calibrated reflectance. If the relative reflectance exceeded 1 at any of the 159 spectral bands, the entire pixel spectrum was classified as an abnormal high-reflectance spectrum and excluded from subsequent spectral aggregation. The entire vector was removed, rather than only the affected wavelength, to avoid retaining a partially artifact-contaminated spectrum in the representative coin-level mean spectrum. The criterion was defined as follows:

(4) $$\underset{\lambda }{\max}R_{i}\left(\lambda \right)>1$$

where $\underset{\lambda }{\max}R_{i}\left(\lambda \right)$ represents the maximum reflectance value of the *i*th hyperspectral vector among the 159 spectral bands. When $\underset{\lambda }{\max}R_{i}\left(\lambda \right)$ is greater than 1, it means that at least one band in this spectral vector has a reflectance value above the defined range. Therefore, this vector was removed from the hyperspectral vector matrix and was not included in the later mean spectrum calculation. In contrast, when the reflectance values of all bands in the vector were less than or equal to 1, the vector was regarded as a valid spectrum and was retained.

After removing the spectra with abnormally high reflectance, assume that *k* valid hyperspectral vectors were retained and denoted as *x*_1_, *x*_2_, …, *x_k_*. The mean spectrum of the coin, *X_mean_*, can then be calculated as follows:

*X_mean_* = (*x*_1_ + *x*_2_ + … + *x_k_*)/*k* (5)

where *X_mean_* is the representative mean spectrum of the coin. It contains the mean reflectance values of 159 spectral bands. *k* represents the number of valid pixels retained after removing abnormal spectra. The above calculation was performed band by band. In other words, the mean reflectance of all valid spectral vectors was calculated separately at each spectral band.

Using this processing workflow, this study first used the ROI mask to extract the hyperspectral vectors of all pixels within the coin region. Then, vectors with a reflectance value greater than 1 in any spectral band were removed as abnormal high-reflectance spectra. Finally, the remaining valid spectral vectors were used to calculate the mean spectrum of the coin. This method reduced the effect of local specular reflection and abnormal high-reflectance pixels on the mean result. Therefore, the obtained mean spectrum was used as the representative coin- or condition-level spectrum for subsequent spectral comparison and feature analysis. The same ROI-selection, spectral preprocessing, abnormal-pixel removal, and spectral aggregation criteria were applied consistently to all hyperspectral images in this study.

#### 2.4.2. Unit of Analysis and Handling of Repeated Measurements

To avoid pseudoreplication, the unit of analysis in this study was defined at the coin level or acquisition-condition level rather than at the individual-pixel level. As described in Section 2.4.1, pixels within each coin ROI were first subjected to spectral preprocessing. Spectral vectors containing a reflectance value greater than 1 in any spectral band were removed as abnormal high-reflectance spectra. The remaining valid pixel spectra were then used to calculate within-ROI spectral variability or to derive a representative mean spectrum for each coin or acquisition condition. These individual valid pixels were treated as components of the spectral aggregation process and were not regarded as independent sample replicates for statistical inference or performance estimation.

For the support-material and rotation-angle experiments, the same physical coin was imaged under multiple controlled acquisition conditions. The resulting measurements were treated as within-coin condition comparisons and were not considered independent coin samples. SAM was calculated between the representative mean spectra obtained for each coin or acquisition condition, rather than between individual pixels. No supervised classification model was developed in the present study; therefore, no training, validation, or test data split was performed, and no classification performance estimates were calculated. Future classification studies should use coin-level data splitting or leave-one-coin-out cross-validation to ensure that pixels or repeated measurements from the same physical coin do not appear in both the training and test sets.

#### 2.4.3. Spectral Angle Difference Analysis

This study used the spectral angle mapper (SAM) to quantify the difference between the mean spectra of different coin samples or different imaging conditions. SAM is a commonly used spectral similarity index in hyperspectral image analysis. It treats each spectrum as a vector in a multi-band spectral space and calculates the angle between two spectral vectors [17]. A smaller SAM value indicates that the two spectra have more similar spectral shapes. A larger SAM value indicates a greater spectral difference. The SAM between two mean spectra was calculated as follows:

(6) $$\mathrm{SAM}\left(X_{\mathrm{mean}}^{\left(m\right)},X_{\mathrm{mean}}^{\left(n\right)}\right)=\cos^{-1}\left(\frac{X_{\mathrm{mean}}^{\left(m\right)}\cdot X_{\mathrm{mean}}^{\left(n\right)}}{\left\|X_{\mathrm{mean}}^{\left(m\right)}\right\|\left\|X_{\mathrm{mean}}^{\left(n\right)}\right\|}\right)$$

where $X_{\mathrm{mean}}^{\left(m\right)}$ and $X_{\mathrm{mean}}^{\left(n\right)}$ are the mean spectral vectors of two coin samples or two imaging conditions to be compared. $X_{\mathrm{mean}}^{\left(m\right)}\cdot X_{\mathrm{mean}}^{\left(n\right)}$ represents the inner product of the two vectors. $\left\|X_{\mathrm{mean}}^{\left(m\right)}\right\|$ and $\left\|X_{\mathrm{mean}}^{\left(n\right)}\right\|$ represent the lengths of the two vectors. In this study, SAM was used to compare spectral differences among different supports, different rotation angles, new and circulated NT$10 coins, different NT$10-coin design variants, and different coin denominations, including a special-year coin. For easier interpretation, SAM values were also converted from radians to degrees. In the present study, SAM was used only as a descriptive pairwise index of spectral difference between representative coin- or acquisition-condition-level mean spectra. SAM values were not interpreted as statistical evidence of class separability, classification accuracy, or recognition performance. Because several spectral comparison categories contained only one independent coin, valid estimates of within-group variability, confidence intervals, standardized effect sizes, and between-group statistical significance could not be obtained without treating individual pixels as independent replicates. Such pixel-level inference was intentionally avoided to prevent pseudoreplication. Therefore, no inferential statistical tests were performed for these category-level spectral comparisons.

## 3. Results

### 3.1. Results of Imaging Position and Aperture Conditions

**Figure 5:**
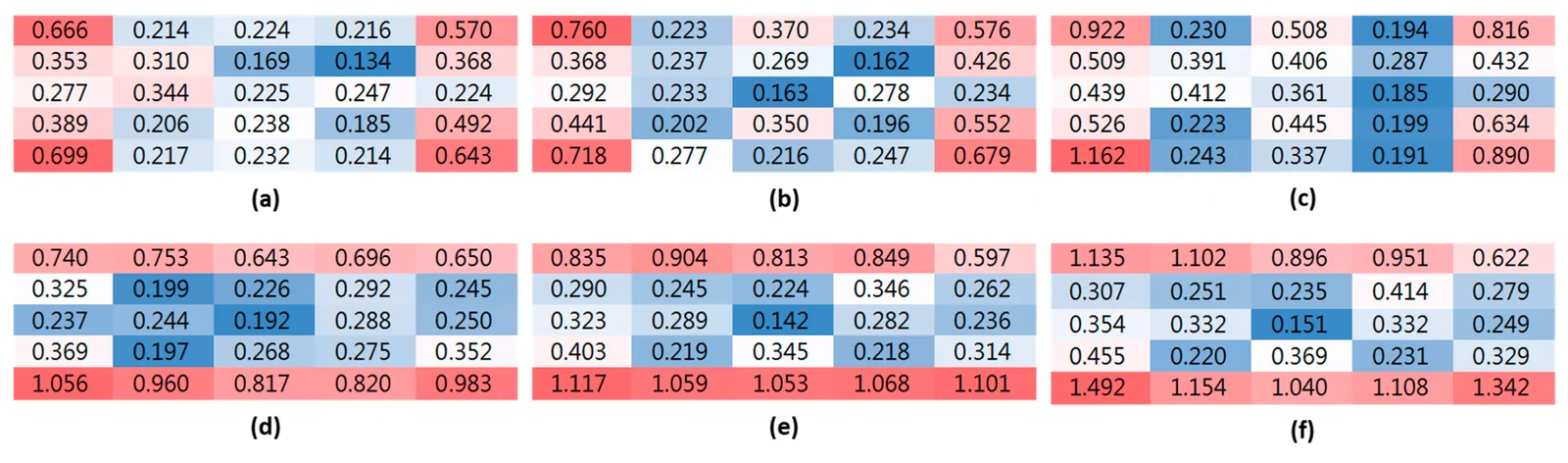
Heatmaps of the mean spectral standard deviation at 25 positions under different coin sides and aperture conditions. (**a**) F1.8_H; (**b**) F2.8_H; (**c**) F4_H; (**d**) F1.8_W; (**e**) F2.8_W; (**f**) F4_W. H represents the obverse side of the coin, namely the portrait side. W represents the reverse side of the coin, namely the side with text and denomination marks. Each value represents the mean standard deviation across all spectral bands within the coin ROI at that imaging position. A lower value indicates smaller spectral variation and better imaging stability. In the heatmaps, blue indicates lower mean spectral standard deviation and greater imaging stability, whereas red indicates higher mean spectral standard deviation and greater spectral variation; intermediate colors represent values between these two extremes.

Figure 5 shows the mean spectral standard deviation across the 25 imaging positions under six combinations of coin side and aperture setting. Lower values indicate lower within-ROI spectral variation. The complete values and position rankings are provided in Supplementary Tables S2 and S3.

For the obverse side, the lowest mean spectral standard deviation occurred at position 9 for F1.8_H (0.134) and F2.8_H (0.162), and at position 14 for F4_H (0.185). Thus, the optimal position was not consistent across aperture settings.

In contrast, position 13 ranked first on the reverse side under all three aperture settings, with mean spectral standard deviations of 0.192, 0.142, and 0.151 for F1.8_W, F2.8_W, and F4_W, respectively. Among these conditions, F2.8_W showed the lowest spectral variation. Table 4 summarizes the corresponding position rankings and mean spectral standard deviations.

Taken together, the obverse-side optimum varied with aperture, whereas position 13 on the reverse side consistently showed the lowest mean spectral standard deviation across all three aperture settings. Because F2.8_W produced the lowest value at position 13, the reverse side, position 13, and F2.8 were selected as the standardized acquisition conditions for subsequent experiments.

### 3.2. Effects of Support Material and Rotation Angle on Spectral Measurement

After the imaging position, coin side, and aperture were standardized, the effects of support material and in-plane coin rotation on SWIR spectral measurement were further evaluated. These experiments were used to determine whether external acquisition conditions could introduce additional spectral variation before subsequent coin comparisons.

**Figure 6:**
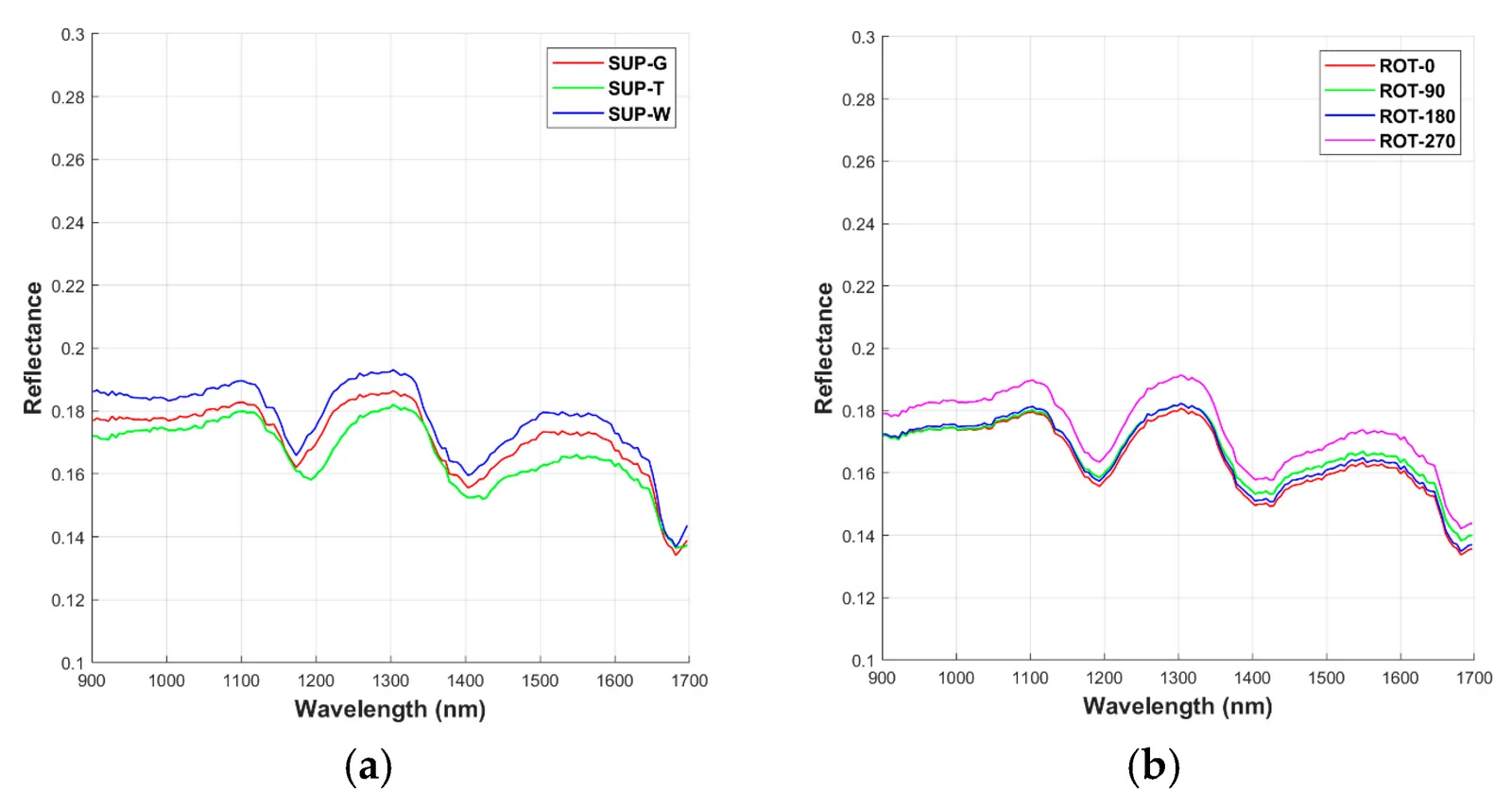
Effects of support material and coin rotation angle on the short-wave infrared reflectance spectra of an NT$10 coin. (**a**) Mean reflectance spectra of the same NT$10 coin placed on the green, transparent, and white supports; (**b**) mean reflectance spectra of the same NT$10 coin at in-plane rotation angles of 0°, 90°, 180°, and 270°.

For the support-material experiment, the same NT$10 coin was imaged on the green, transparent, and white supports. As shown in Figure 6a, the three conditions produced similar overall spectral profiles, but differences in reflectance intensity were observed. The white support produced the highest overall reflectance, whereas the transparent support produced the lowest, with the green support showing intermediate values. These differences were observed even though the analysis was restricted to the coin ROI. Pairwise SAM values ranged from 0.232 to 1.087 (Table 5). The smallest difference was observed between the green and white supports (SAM = 0.232), whereas the largest was observed between the transparent and white supports (SAM = 1.087). These findings indicate that support material can introduce measurable variation in the recorded coin spectrum. Therefore, the transparent support was fixed for all subsequent single-coin experiments to maintain a consistent support condition.

For the rotation-angle experiment, the same NT$10 coin was imaged at 0°, 90°, 180°, and 270°. As shown in Figure 6b, the four spectra retained similar overall profiles, although the 270° condition showed slightly higher reflectance than the other orientations. Pairwise SAM values ranged from 0.132 to 0.581 (Table 6). The smallest spectral difference was observed between 0° and 180° (SAM = 0.132), whereas the largest was observed between 0° and 90° (SAM = 0.581). Thus, measurable spectral variation was observed when the same coin was rotated while the other acquisition conditions remained fixed.

Overall, both support material and coin orientation affected the measured SWIR spectra of the same NT$10 coin. These results support controlling both factors during subsequent spectral comparisons. Accordingly, the transparent support and a fixed coin orientation were used in all later single-coin experiments. The observed orientation-dependent differences may reflect changes in the interaction among illumination, surface relief, and directional reflection; however, the individual optical contributions were not independently quantified in this study.

### 3.3. Spectral Differences Between New and Circulated NT$10 Coins

**Figure 7:**
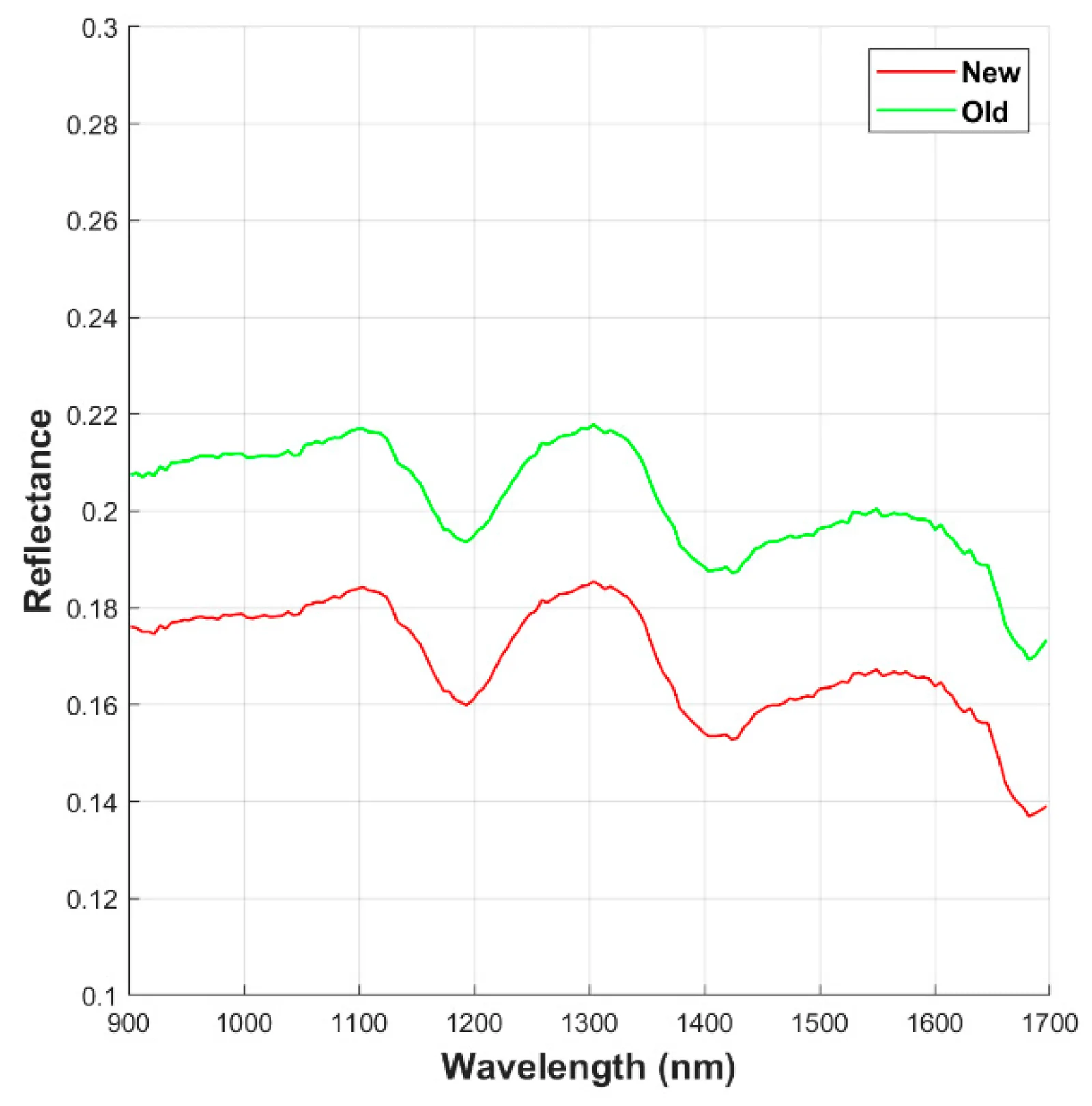
Mean SWIR reflectance spectra of the selected new and circulated NT$10 coins acquired under the standardized imaging conditions.

Under the standardized imaging conditions, the selected new and circulated NT$10 coins were compared using their representative mean SWIR reflectance spectra. As shown in Figure 7, the two coins exhibited similar overall spectral profiles in the 900–1700 nm range, with reflectance decreases near 1180 nm, 1400 nm, and 1700 nm. However, the circulated coin showed higher reflectance than the new coin over most spectral bands.

SAM was used to quantify the spectral difference between the two representative mean spectra. The SAM value was 0.729, indicating a measurable spectral difference between the selected new and circulated NT$10 coins under the standardized imaging conditions.

These results show that the selected new and circulated NT$10 coins exhibited a quantifiable spectral difference under the standardized imaging conditions used in this study. However, this comparison was based on representative samples and should not be interpreted as validated surface-condition assessment.

Because the two coins represented different documented surface-use conditions, the observed difference should be regarded as a sample-specific spectral difference. Any attribution of this difference to wear, oxidation, contamination, or surface-preservation state would require independent surface characterization.

### 3.4. Spectral Differences Among Different NT$10 Coin Design Variants

**Figure 8:**
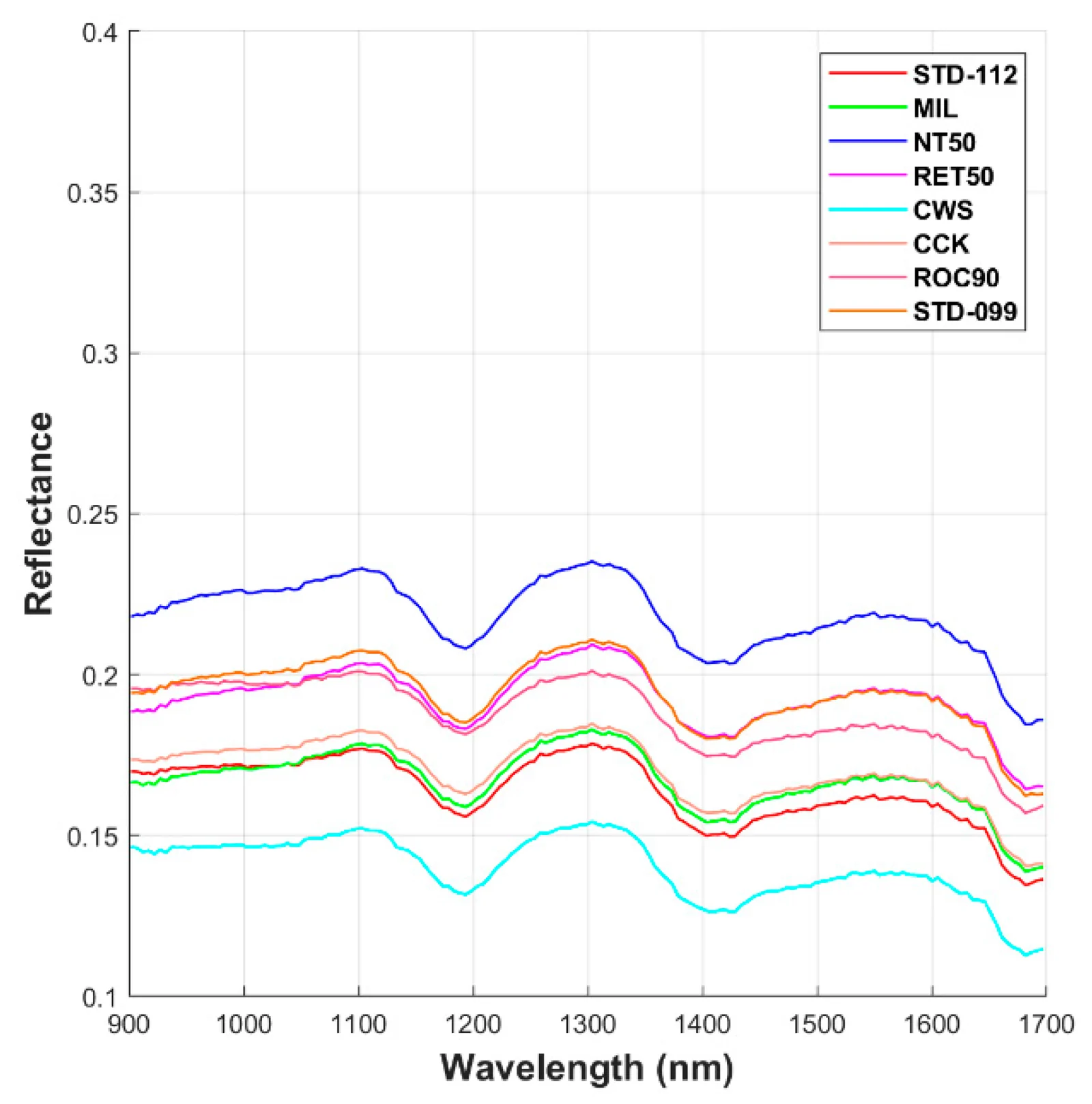
Mean SWIR reflectance spectra of the eight selected NT$10-coin design variants acquired under the standardized imaging conditions.

Eight NT$10-coin design variants were compared under the standardized acquisition conditions. The sample codes and descriptions are summarized in Table 7. As shown in Figure 8, the eight samples exhibited similar overall spectral profiles in the 900–1700 nm range, with reflectance decreases mainly near 1180 nm and 1400 nm and another decrease near 1700 nm. However, differences in overall reflectance intensity were observed among the selected variants. NT50 showed the highest overall reflectance, whereas CWS showed the lowest.

Pairwise SAM values ranged from 0.260 to 1.680 (Table 8). The smallest spectral difference was observed between NT50 and STD-099 (SAM = 0.260), followed by MIL and STD-099 (SAM = 0.294). The largest difference was observed between RET50 and CWS (SAM = 1.680). These results indicate that the selected NT$10 design variants showed measurable differences in both reflectance intensity and spectral shape under the controlled imaging conditions.

Because the selected variants also differed in characteristics such as design, year of issue, relief structure, and surface-use history, the present data do not allow the contribution of these individual factors to be separated. Therefore, the observed differences should be interpreted as descriptive spectral differences among the selected design-variant samples rather than as evidence of a specific material or surface-related cause.

### 3.5. Spectral Differences Among Different Coin Denominations and a Special-Year Coin

To descriptively compare the spectral differences among the selected coin denominations, this study further compared the short-wave infrared reflectance spectra of six denomination or special-year coin samples. The sample codes and English descriptions of the selected samples are summarized in Table 9.

**Figure 9:**
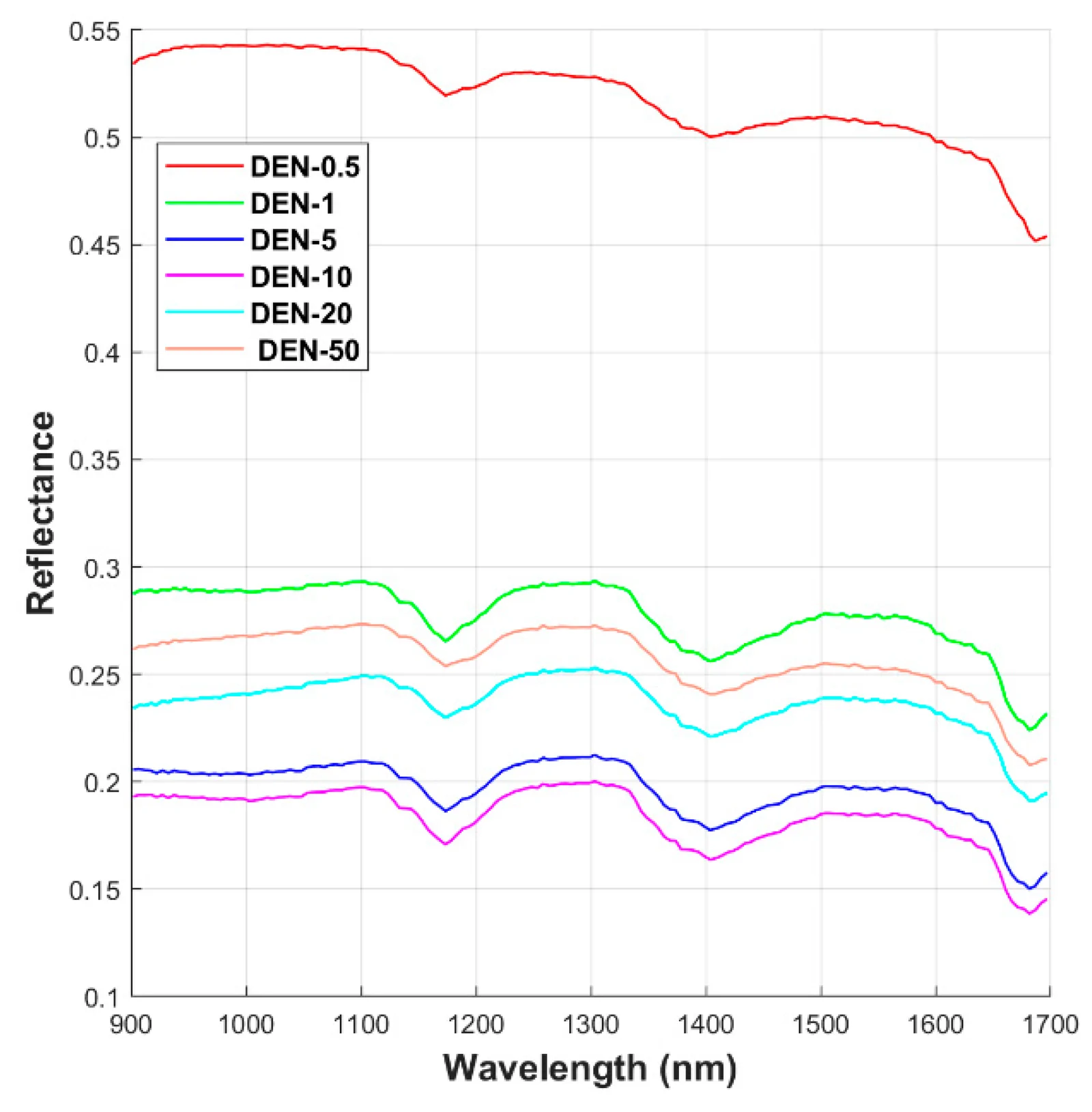
Mean SWIR reflectance spectra of the six selected coin denomination or special-year samples acquired under the standardized imaging conditions.

As shown in Figure 9, the six samples exhibited similar overall spectral profiles in the 900–1700 nm range, with reflectance decreases near 1180 nm and 1400 nm and another decrease near 1700 nm. However, differences in overall reflectance intensity were observed among the samples.

Among the selected samples, DEN-0.5 showed the highest overall reflectance, while DEN-1 and DEN-50 showed relatively higher reflectance than the remaining current-denomination samples. DEN-5 and DEN-10 showed relatively lower reflectance, and DEN-10 had the lowest reflectance in most spectral bands.

Pairwise SAM values ranged from 0.458 to 2.449 (Table 10). The smallest spectral difference was observed between DEN-5 and DEN-10 (SAM = 0.458), whereas the largest was observed between DEN-0.5 and DEN-10 (SAM = 2.449). These results indicate that the selected denomination and special-year samples showed measurable differences in both reflectance intensity and spectral shape under the controlled imaging conditions.

It should be noted that this study did not perform elemental-composition or independent surface-characterization analyses. Therefore, the spectral differences among different coin denominations were not interpreted as evidence of specific alloy composition or other individual physical causes. The observed differences should instead be regarded as overall spectral differences associated with the selected coin samples under the controlled imaging conditions.

### 3.6. Initial Repeatability Evaluation Under Standardized Acquisition Conditions

An initial repeatability evaluation was performed under the current standardized experimental conditions using three independent NT$10 coins across two measurement days, two operators, and repeated coin repositioning, resulting in a total of 24 hyperspectral acquisitions. The repositioning comparisons (*n* = 12) yielded a mean pairwise SAM of 0.2450 ± 0.1485°, with a range of 0.0799–0.6371°. The inter-day comparisons (*n* = 6) showed a mean SAM of 0.6368 ± 0.1913° (range, 0.4494–1.0085°), whereas the inter-operator comparisons (*n* = 6) showed a mean SAM of 0.3323 ± 0.1550° (range, 0.1238–0.5655°). Among the three evaluated sources of variation, the inter-day comparisons showed the largest mean spectral angle difference.

For reflectance-magnitude repeatability, the coin-level median spectral CVs calculated across the eight acquisitions were 0.74%, 0.83%, and 0.95% for C1, C2, and C3, respectively. The overall median spectral CV across the three independent coins was 0.83%, with a range of 0.74–0.95%. Thus, all three coin-level median spectral CV values were below 1%. The results of this initial repeatability evaluation are summarized in Table 11.

## 4. Discussion

### 4.1. Principal Findings of This Study

This study established a workflow for non-destructive spectral analysis of coins using 900–1700 nm short-wave infrared hyperspectral imaging (SWIR-HSI). It also systematically evaluated the effects of imaging position, aperture setting, coin side, support material, and rotation angle on spectral measurement. The main results showed that, in the experiment where 25 NT$10 coins were imaged at the same time, the reverse side at position 13 showed the lowest mean spectral standard deviation under all three aperture conditions, F1.8, F2.8, and F4. Among them, the F2.8 condition had the lowest mean spectral standard deviation. Therefore, the reverse side, position 13, and F2.8 were selected as the standardized imaging conditions for later spectral comparisons.

The additional repeatability experiment provided an initial quantitative evaluation of measurement repeatability under the current standardized experimental conditions. Mean SAM values were 0.2450°, 0.6368°, and 0.3323° for repositioning, inter-day, and inter-operator comparisons, respectively, while the overall median spectral CV was 0.83% (coin-level range, 0.74–0.95%). Among the evaluated sources, inter-day measurements showed the largest spectral angle variation. These findings indicate that measurement variability remained quantifiable even under standardized conditions and that day-to-day acquisition represents an important source of residual variation. Accordingly, the repeatability findings should be interpreted as an initial assessment under the present experimental conditions rather than as comprehensive validation of the standardized protocol.

In addition, this study found that support material and coin rotation angle both caused measurable spectral differences. This indicates that external imaging conditions have an important effect on SWIR reflectance measurement of metallic coins. Under standardized conditions, new and circulated NT$10 coins, different NT$10 coin design variants, and different coin denominations, including a special-year coin, showed different levels of spectral differences. These differences could be further quantified using the SAM. However, the magnitude of many pairwise SAM differences was limited, and these values should not be interpreted as evidence of strong group separation or classification-level differentiation. Overall, this study shows that, after imaging standardization, SWIR-HSI can provide quantifiable spectral differences among selected coins with different surface-use conditions, design variants, and denominations. These differences are descriptive and should not be interpreted as evidence of a specific underlying physical or chemical mechanism.

### 4.2. Importance of Standardizing SWIR-HSI Imaging Conditions for Coins

Coins are highly reflective metallic samples. Their surfaces also contain text, patterns, edges, and relief structures. Therefore, their spectral measurements can be easily affected by illumination angle, camera position, aperture setting, directional surface reflection, and background support. Previous studies on ancient coin image recognition have also reported that coin classification can be affected by preservation condition, pattern position changes, in-plane rotation, uneven lighting, and background interference [2]. This shows that coin image analysis has high geometric and surface variability. Therefore, if imaging conditions are not controlled first, the observed spectral differences may include both the differences from the coins themselves and the variation caused by measurement conditions. Among these acquisition factors, illumination configuration is especially important for metallic coins because the measured signal can change substantially with the direction of incident light and specular reflection from local relief structures. In the present study, the four halogen lamps were therefore mounted with their optical axes directed vertically downward, approximately parallel to the camera optical axis and normal to the sample plane. The lamps were not tilted inward toward the coin because such directional illumination produced strong reflected signals from the metallic surface. This illumination geometry was kept fixed throughout all experiments and was treated as a primary acquisition control. However, illumination angle was not systematically varied as an independent experimental factor in the present study. Future studies should evaluate different illumination angles and geometries to determine their effects on spectral repeatability more quantitatively.

In the preliminary experiment, this study found that the reverse side of the coin showed the most stable result at position 13 under all three aperture settings. This suggests that the center position of the image field may provide more consistent measurement conditions. This result may be related to the fact that the center field is less affected by lens edge effects, uneven lighting, or changes in geometric reflection. In addition, F2.8 showed the lowest mean spectral standard deviation at position 13 on the reverse side. This suggests that, under the imaging setup used in this study, F2.8 may provide a better balance among light input, depth of field, and spectral stability. It should be emphasized that this study does not claim that F2.8 is the best aperture in all situations. Instead, it shows that, under the SWIR-HSI setup and coin imaging conditions used in this study, the combination of the reverse side, position 13, and F2.8 had better stability across different conditions.

The support-material and rotation-angle experiments further supported the need for imaging standardization. Although the overall spectral profiles of the same NT$10 coin remained generally similar, support material produced measurable differences in reflectance intensity and SAM values, and different in-plane rotation angles also resulted in quantifiable spectral differences. The surrounding support could potentially influence the local optical environment through scattering, edge reflection, or background reflection. Likewise, the spectral differences observed at different in-plane rotation angles may reflect changes in the interaction between illumination, surface relief, and directional reflection. However, these optical contributions were not independently measured in the present study and therefore should not be interpreted as confirmed physical mechanisms.

Although HSI provides both spatial and spectral information, the present rotation-angle experiment used the representative mean spectrum as an acquisition-condition-level descriptor because the primary objective was to determine whether coin orientation affected the overall spectral measurement under otherwise fixed conditions. A spatially resolved comparison among rotated hyperspectral images would require geometric registration of corresponding surface features before pixelwise or local spectral differences could be meaningfully interpreted. Without such registration, apparent spatial differences could arise simply because different relief structures, text, or local regions occupy different pixel locations after rotation. Therefore, spatially resolved rotation analysis was beyond the scope of the present acquisition-standardization study. Future studies should investigate registered spectral-difference maps, local SAM maps, spatial variability, texture features, or other spatial–spectral descriptors to more fully utilize the imaging information provided by HSI.

These results indicate that, for metallic coins, imaging conditions themselves are important factors that affect spectral measurement. Therefore, fixing the coin side, imaging position, aperture, support, and rotation angle is necessary before making reliable spectral comparisons.

### 4.3. Spectral Differences Related to Surface Condition and NT$10 Coin Design Variants

Under the standardized imaging conditions, the new and circulated NT$10 coins showed similar main spectral patterns. However, their overall reflectance intensity and SAM values showed that there were still quantifiable differences between them. The circulated NT$10 coin showed higher reflectance in most spectral bands. Several factors, including surface wear, small scratches, oxidation, contamination, surface roughness, and reflection behavior, could potentially contribute to this difference. However, the present measurements cannot distinguish the contribution of any individual factor. Therefore, the spectral difference between the new and circulated coins observed in this study should be interpreted as an overall spectral feature related to surface use condition. It should not be interpreted as the result of a single material component or a single physical mechanism.

A similar finding was observed for different NT$10-coin design variants. Although the eight NT$10-coin design variants showed similar main spectral patterns, their overall reflectance intensity and SAM differences could still be quantified. The selected design variants also differed in several characteristics, including design pattern, relief structure, year of issue, surface preservation condition, degree of wear, and potentially other material or surface properties. The present data do not allow the contribution of these factors to be separated. Therefore, the observed SAM and reflectance differences should be interpreted as overall descriptive spectral differences among the selected design-variant samples rather than as evidence of a specific material or surface cause.

These findings are consistent with the use of hyperspectral imaging in cultural heritage and multi-material surface analysis. Previous studies have reported that multispectral and hyperspectral imaging can be used as non-invasive methods for spectral recording, material mapping, and surface condition analysis of large objects [5,6]. This study extends this concept to metallic coin samples. The results show that even for highly reflective coins with complex surface structures, SWIR-HSI can still provide meaningful spectral information for comparison when the imaging conditions are standardized.

### 4.4. Spectral Differences Among Different Coin Denominations and Methodological Meaning

The results for different coin denominations and the special-year coin showed that the main spectral patterns were generally similar among the coin groups. However, the differences in overall reflectance intensity and SAM values were more obvious. In particular, DEN-0.5 showed higher reflectance and larger SAM differences compared with the other current circulating coin denominations. Among the selected samples, DEN-0.5 showed relatively larger descriptive spectral differences from several of the other denomination samples. In contrast, DEN-5 and DEN-10 had the lowest SAM value, indicating that their spectral shapes were more similar. However, these pairwise SAM differences should not be interpreted as evidence of statistical class separability because only one representative coin was included for each denomination category. The denomination samples also differed in several characteristics, including year, material, surface condition, size, and relief structure. These factors may all contribute to the measured spectra; however, the present data cannot determine their individual contributions. Therefore, the observed differences should be interpreted as descriptive spectral differences among the selected coin samples rather than as evidence of a specific physical cause.

The value of this study is not to claim that SWIR-HSI can directly replace elemental analysis methods. Instead, this study establishes a non-contact, non-destructive, and standardized workflow for spectral feature analysis of coins. Traditional studies on coin composition or corrosion often use methods such as XRF, PIXE, SEM-EDS, or Raman spectroscopy to analyze elements and corrosion products. These methods can provide more direct material or chemical information, but they are mostly local or point-based analysis methods [3,4]. In comparison, SWIR-HSI can rapidly obtain the mean spectrum and spatial distribution information from the whole coin ROI under the same imaging conditions. In the present study, this capability was used for standardized spectral comparison rather than for automatic coin classification or validated surface-condition assessment.

This research direction is also consistent with HSI applications in banknotes and anti-counterfeiting materials. Previous studies used push-broom HSI to build a spectral library of polymer banknotes and used principal component analysis (PCA) to develop classification criteria. Related reviews have also reported that HSI has been applied to anti-counterfeiting and identification studies of documents, artworks, photographs, holograms, and currency [7,8]. The difference between this study and previous studies is that the target samples in this study were metallic coins, rather than banknotes or document materials. Therefore, additional issues needed to be considered, including high reflectance from metal surfaces, relief structures, rotation angle, and support effects. This is also why imaging standardization was treated as a key step in this study.

### 4.5. Limitations and Future Work

This study has several limitations. First, although 25 independent new and uncirculated NT$10 coins were included in the imaging-position and aperture optimization experiment, the sample size and category coverage of the subsequent spectral comparison experiments were limited. The new-versus-circulated comparison included one coin for each surface-use condition, the design-variant comparison included one independent coin for each of eight design variants, and the denomination comparison included one independent coin for each of six denomination or special-year categories. Therefore, the observed spectral differences should be interpreted as preliminary and sample-specific findings under the controlled imaging conditions used in this study. They should not be generalized to all coins of the same denomination, design variant, year, or surface condition. For the same reason, statistical significance, confidence intervals, and standardized effect sizes were not calculated for these category-level comparisons because the required independent within-group replication was not available. Treating individual ROI pixels as independent observations for these analyses would result in pseudoreplication. Future studies should include multiple independent coins within each category, broader year coverage, and predefined levels of wear and surface condition to evaluate within-group and between-group variability.

Second, SAM quantifies spectral dissimilarity but does not identify the physical or chemical origin of that dissimilarity. This study did not perform X-ray fluorescence (XRF), scanning electron microscopy–energy dispersive X-ray spectroscopy (SEM-EDS), Raman spectroscopy, or surface roughness measurement. Therefore, it was not possible to directly determine whether the spectral differences were caused by specific alloy composition, oxides, contaminants, or surface morphology changes. Previous studies on metal corrosion have shown that 900–1700 nm SWIR-HSI can be used to identify corrosion minerals on carbon steel surfaces. It can also be combined with reference spectra or other reference methods for material analysis [14]. Accordingly, future studies that aim to relate SWIR spectral differences to material composition, oxidation, or surface preservation should include independent reference measurements, such as XRF, SEM-EDS, Raman spectroscopy, microscopic analysis, or surface-roughness measurement. These measurements would allow the spectral findings to be compared with independently characterized material and surface properties.

Third, the optimization of imaging position and aperture setting in this study was performed using 25 NT$10 coins placed on a white support at the same time. However, the later single-coin comparison experiments were performed using the transparent support. Although all later single-coin comparisons were completed under the same transparent support condition, and the support effect was fixed across groups, this study showed that support material can affect spectral measurement. Therefore, future studies should repeat the imaging position and aperture optimization under the transparent support condition. This can confirm whether the best imaging position and aperture setting remain consistent under different support conditions.

Fourth, the additional repeatability experiment provided an initial quantitative assessment of repositioning, inter-day, and inter-operator variability under the standardized acquisition workflow. The mean SAM values were 0.2450°, 0.6368°, and 0.3323° for repositioning, inter-day, and inter-operator comparisons, respectively, and the overall median spectral CV was 0.83% (coin-level range, 0.74–0.95%). Although the coin-level median spectral CV values were all below 1%, inter-day comparisons showed the largest spectral angle variation among the three evaluated sources.

This repeatability experiment used the same SWIR-HSI instrument and the same fixed gantry configuration. Different measurement days involved independent system startup, lamp warm-up, and calibration procedures, but complete disassembly and reconstruction of the imaging setup and inter-instrument variability were not evaluated. Accordingly, the present results should be regarded as an initial repeatability evaluation under the current experimental conditions and should not be interpreted as broader protocol validation. In addition, residual variation in illumination output, detector dark signal, manual ROI delineation, local specular reflection, and other acquisition-related factors may still contribute to measurement uncertainty. Although the standardized acquisition and calibration procedures used in this study were designed to reduce these effects, they cannot be assumed to eliminate them completely. These factors should be investigated in future protocol-validation studies.

Finally, although HSI provides both spatial and spectral information, the present study primarily used representative mean spectra and SAM for condition-level spectral comparisons. Spatially resolved analyses of rotated coin images, including image registration and local spatial–spectral metrics, were not performed and should be investigated in future studies. A complete supervised classification model was not established. Accordingly, no training, validation, or test data split was performed in the present study. After sufficiently large independent coin datasets have been collected, future studies may apply methods such as principal component analysis (PCA), partial least squares-discriminant analysis (PLS-DA), support vector machine (SVM), or random forest to evaluate classification performance. Such studies should use coin-level data splitting or leave-one-coin-out cross-validation and should include appropriate comparisons with RGB or visible-light imaging, point spectroscopy, non-standardized HSI workflows, and conventional spectral analysis methods. This can avoid data leakage caused by pixels from the same coin appearing in both the training and testing datasets. These extensions may further clarify the potential of standardized SWIR-HSI for non-destructive coin analysis.

## 5. Conclusions

This study established a standardized workflow for non-destructive spectral difference analysis of coins using 900–1700 nm short-wave infrared hyperspectral imaging (SWIR-HSI). It also systematically evaluated the effects of imaging position, aperture setting, coin side, support material, and rotation angle on spectral measurement. The results showed that, under the imaging conditions used in this study, the reverse side of the coin at position 13 with an F2.8 aperture provided more stable spectral measurements. At the same time, support material and coin rotation angle both caused quantifiable spectral variation. These findings indicate that image acquisition conditions must be controlled before reliable comparisons among metallic coin samples can be performed using SWIR-HSI. An initial repeatability evaluation under the current experimental conditions, using three independent NT$10 coins and 24 hyperspectral acquisitions yielded mean SAM values of 0.2450°, 0.6368°, and 0.3323° for repositioning, inter-day, and inter-operator comparisons, respectively, with an overall median spectral CV of 0.83%. Inter-day measurements showed the largest spectral angle variation among the three evaluated sources.

Under the standardized imaging conditions, this study further observed that new and circulated NT$10 coins, different NT$10-coin design variants, and different coin denominations, including a special-year coin, all showed different levels of short-wave infrared spectral differences. These differences could be quantified using the SAM. These results show that selected coin samples with different surface-use conditions, design variants, and denominations exhibited quantifiable SWIR spectral differences under the controlled imaging conditions used in this study. Although this study did not include elemental composition analysis or surface morphology analysis, and therefore did not directly attribute the spectral differences to specific alloy composition or a single material mechanism, the results demonstrate the feasibility of standardized SWIR-HSI as a non-contact and non-destructive tool for spectral feature analysis of coins.

The main contribution of this study is the evaluation and control of acquisition factors that can affect SWIR-HSI measurements of highly reflective metallic coins. The present results support the use of standardized SWIR-HSI for controlled spectral difference analysis under the imaging conditions used in this study. However, these findings should not be interpreted as evidence of validated coin recognition, automatic classification, or reliable surface-condition assessment. Further studies with larger independent sample sets, additional measurement sessions, complete reconstruction of the imaging setup, inter-instrument validation, and independent material or surface characterization will be required before broader applications can be established.

## Figures and Tables

**Table 1:** Coin samples, support conditions, and experimental design.

Experiment	Sample Used	No. of Independent Coins	Support Condition	Imaging Position	Aperture	Main Purpose
Imaging position and aperture optimization	NT$10 coins	25	Fixed support	25 positions	F1.8, F2.8, F4	To determine the standardized imaging position and aperture
Support material comparison	Same NT$10 coin	1	Green, transparent, and white supports	Position 13	F2.8	To evaluate the effect of support material on spectral measurement
Rotation angle comparison	Same NT$10 coin	1	Transparent support	Position 13	F2.8	To evaluate the directional effects of 0°, 90°, 180°, and 270° rotations
New and circulated coin comparison	One new and one circulated NT$10 coin	2	Transparent support	Position 13	F2.8	To evaluate spectral differences related to surface condition
Design variant comparison	Eight NT$10-coin design variants	8	Transparent support	Position 13	F2.8	To evaluate spectral differences related to design variants and surface condition
Denomination comparison	NT$0.5, NT$1, NT$5, NT$10, NT$20, and NT$50	6	Transparent support	Position 13	F2.8	To evaluate spectral differences among different coin denominations

**Table 2:** Materials and uses of the 3D-printed supports.

Support Code	Support Color	Material	Fabrication Method	Purpose	Used in Subsequent Experiments
SUP-G	Green	Polylactic acid (PLA)	3D printing	To evaluate the effect of support material on coin spectral measurement	No
SUP-T	Transparent	VisiJet M3 Crystal (MJP)	3D printing	To evaluate the effect of support material on coin spectral measurement and to control the coin imaging surface height as the standard support	Yes
SUP-W	White	Polylactic acid (PLA)	3D printing	To evaluate the effect of support material on coin spectral measurement	No

**Table 3:** Short-wave infrared hyperspectral imaging system and imaging parameters.

Parameter	Setting
Hyperspectral imaging system	SWIR-IGA-I, ISUZU OPTICS Co., Ltd., Hsinchu, Taiwan
Sensor type	OW1.7-VS-CL-640 InGaAs detector (Raptor Photonics Ltd., Larne, Northern Ireland, UK)
Spectral range	900–1700 nm
Number of spectral bands	512 spectral bands
Spectral resolution	5 nm using a 30-μm slit
Scanning mode	Push-broom line-scan
Lens	Kowa LM12HC-VIS-SW lens (Kowa Optronics Co., Ltd., Nagoya, Aichi, Japan; focal length: 12 mm; maximum aperture: F1.8)
Aperture setting	F1.8, F2.8, and F4; fixed at F2.8 in subsequent experiments
Light source	Four 50-W halogen lamps (DECOSTAR 51 ALU, OSRAM GmbH, Munich, Germany)
Illumination configuration	Vertically downward; approximately parallel to camera optical axis; no inward tilt toward sample
Imaging distance	30.5 cm
Exposure time	55 ms
White-reference standard	WC-430 99% Spectralon calibrated white plate (ISUZU OPTICS Co., Ltd., Hsinchu, Taiwan), 250–2500 nm
Image calibration	Dark-current and white-reference calibration according to Equation (1)
Standard support	Transparent 3D-printed support
Standard imaging position	Position 13

**Table 4:** Summary of the best imaging positions under different coin sides and aperture conditions.

Imaging Condition	Best Position	Lowest Mean Spectral Standard Deviation	Mean Spectral Standard Deviation at Position 13	Ranking of Position 13
F1.8_H	9	0.134	0.225	11
F2.8_H	9	0.162	0.163	2
F4_H	14	0.185	0.361	11
F1.8_W	13	0.192	0.192	1
F2.8_W	13	0.142	0.142	1
F4_W	13	0.151	0.151	1

**Table 5:** SAM difference matrix of the mean spectra of an NT$10 coin under different support materials.

	SUP-G	SUP-T	SUP-W
SUP-G	0.000	-	-
SUP-T	0.983	0.000	-
SUP-W	0.232	1.087	0.000

**Table 6:** SAM difference matrix of the mean spectra of an NT$10 coin under different rotation angles.

	ROT-0	ROT-90	ROT-180	ROT-270
ROT-0	0.000	-	-	-
ROT-90	0.581	0.000	-	-
ROT-180	0.132	0.512	0.000	-
ROT-270	0.350	0.428	0.284	0.000

**Table 7:** Different NT$10-coin design variants used in this study.

Code	English Description	Coin Type	Imaging Side
STD-112	Standard NT$10 coin, ROC Year 112	Standard circulating coin	Reverse
MIL	Millennium commemorative NT$10 coin	Commemorative circulating coin	Reverse
NT50	50th Anniversary of the Issue of the New Taiwan Dollar commemorative NT$10 coin	Commemorative circulating coin	Reverse
RET50	50th Anniversary of Taiwan Retrocession commemorative NT$10 coin	Commemorative circulating coin	Reverse
CWS	Chiang Wei-shui commemorative NT$10 coin	Commemorative circulating coin	Reverse
CCK	Chiang Ching-kuo centennial commemorative NT$10 coin	Commemorative circulating coin	Reverse
ROC90	90th Anniversary of the Founding of the Republic of China commemorative NT$10 coin	Commemorative circulating coin	Reverse
STD-099	Standard NT$10 coin, ROC Year 99	Standard circulating coin	Reverse

**Table 8:** SAM difference matrix of the mean spectra of different NT$10-coin design variants.

	STD-112	MIL	NT50	RET50	CWS	CCK	ROC90	STD-099
STD-112	0.000	-	-	-	-	-	-	-
MIL	0.958	0.000	-	-	-	-	-	-
NT50	0.844	0.519	0.000	-	-	-	-	-
RET50	1.453	0.595	0.701	0.000		-	-	-
CWS	0.400	1.135	1.147	1.680	0.000	-	-	-
CCK	0.375	0.648	0.542	1.124	0.693	0.000	-	-
ROC90	0.543	1.191	0.879	1.543	0.908	0.595	0.000	-
STD-099	0.797	0.294	0.260	0.672	1.042	0.475	0.963	0.000

STD-112 = Standard NT$10 coin, ROC Year 112; MIL = Millennium commemorative NT$10 coin; NT50 = 50th Anniversary of the Issue of the New Taiwan Dollar commemorative NT$10 coin; RET50 = 50th Anniversary of Taiwan Retrocession commemorative NT$10 coin; CWS = Chiang Wei-shui commemorative NT$10 coin; CCK = Chiang Ching-kuo centennial commemorative NT$10 coin; ROC90 = 90th Anniversary of the Founding of the Republic of China commemorative NT$10 coin; STD-099 = Standard NT$10 coin, ROC Year 99.

**Table 9:** Coin denomination groups used in this study.

Code	English Description	Coin Group	Imaging Side
DEN-0.5	NT$0.5 coin, ROC Year 38	Special-year coin	Reverse
DEN-1	NT$1 coin	Current circulating denomination	Reverse
DEN-5	NT$5 coin	Current circulating denomination	Reverse
DEN-10	NT$10 coin	Current circulating denomination	Reverse
DEN-20	NT$20 coin	Current circulating denomination	Reverse
DEN-50	NT$50 coin	Current circulating denomination	Reverse

**Table 10:** SAM difference matrix of the mean spectra of different coin denominations and a special-year coin.

	DEN-0.5	DEN-1	DEN-5	DEN-10	DEN-20	DEN-50
DEN-0.5	0.000	-	-	-	-	-
DEN-1	1.373	0.000	-	-	-	-
DEN-5	2.102	0.782	0.000	-	-	-
DEN-10	2.449	1.118	0.458	0.000	-	-
DEN-20	1.681	0.933	1.137	1.545	0.000	-
DEN-50	1.317	0.669	0.983	1.365	0.894	0.000

**Table 11:** Initial repeatability evaluation of the standardized SWIR-HSI acquisition workflow across coin repositioning, measurement days, and operators under the current experimental conditions.

Variability Source	No. of Comparisons	SAM, Mean ± SD (°)	SAM Range (°)	Median Spectral CV (%)
Repositioning	12	0.2450 ± 0.1485	0.0799–0.6371	—
Inter-day	6	0.6368 ± 0.1913	0.4494–1.0085	—
Inter-operator	6	0.3323 ± 0.1550	0.1238–0.5655	—
Overall repeatability, C1	8 acquisitions	—	—	0.74
Overall repeatability, C2	8 acquisitions	—	—	0.83
Overall repeatability, C3	8 acquisitions	—	—	0.95
Overall spectral CV	3 coins	—	—	0.83 (0.74–0.95)

## Data Availability

The data presented in this study are available on request from the corresponding author due to responsible-use and legal considerations related to the public release of complete image datasets of real currency objects. The complete raw hyperspectral image datasets are not publicly available because they contain detailed image information of New Taiwan dollar coin surfaces and associated acquisition files. Access to the data may be provided for academic research purposes upon reasonable request and after review by the corresponding author. The processed data supporting the main findings are included in the article and Supplementary Materials.

## Supplementary Materials

The following supporting information can be downloaded at: https://www.mdpi.com/article/10.3390/s26196264/s1, Table S1: Detailed information on the independent coin samples used in the six primary experiments; Table S2: Complete mean spectral standard deviations at 25 positions under different coin sides and aperture conditions Supplementary Table S2 lists the complete mean spectral standard deviations at 25 positions under six imaging conditions: F1.8_H, F2.8_H, F4_H, F1.8_W, F2.8_W, and F4_W; Table S3: Stability ranking of 25 positions under different coin sides and aperture conditions Supplementary Table S3 lists the ranking results of the 25 positions under the six imaging conditions, sorted from the lowest to the highest mean spectral standard deviation. A higher ranking indicates lower spectral variation and better imaging stability at that position; Figure S1: Actual coin samples used in the different SWIR-HSI experiments.
