# Supplementary material for: Standardized Short-Wave Infrared Hyperspectral Imaging for Non-Destructive Spectral Comparison of Coin Denominations, Design Variants, and Surface-Use Conditions
Source: Sensors (Basel). 2026 Oct 2;26(19):6264. doi: 10.3390/s26196264 (PMC13667621; doi:10.3390/s26196264)
Supplement: Supplementary file 1 [file sensors-26-06264-s001.zip › sensors-4484248-supplementary.pdf]

Supplementary Figure S1

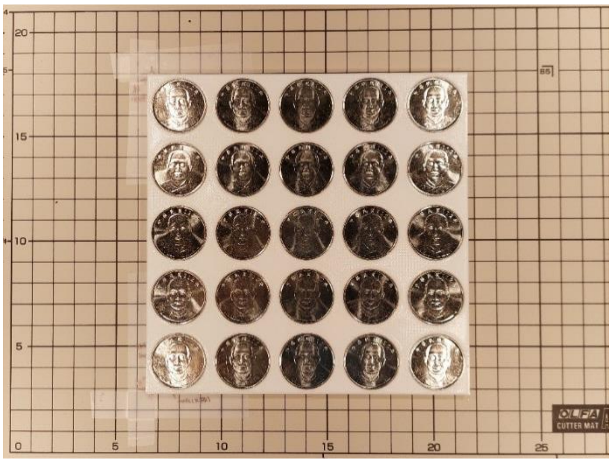

(a)

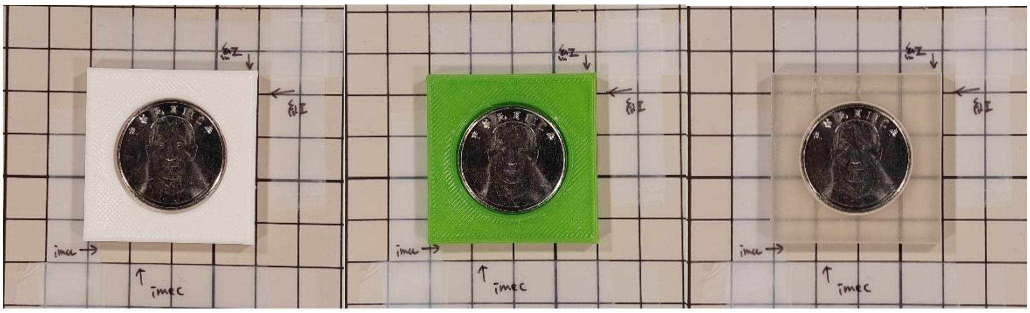

(b)

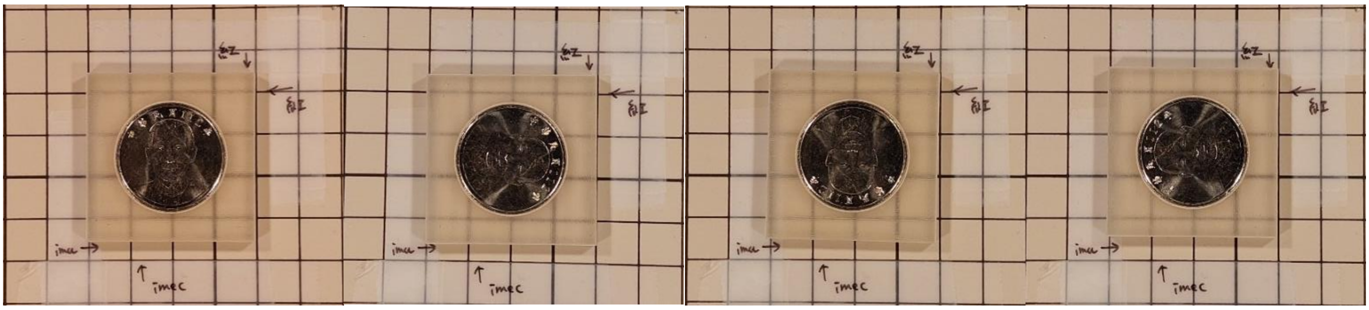

(c) 0°→90°→180°→270°

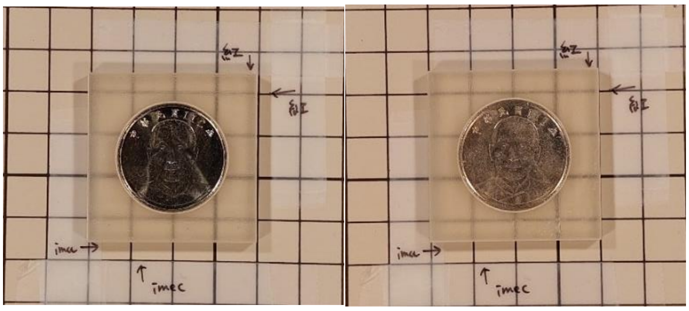

(d)

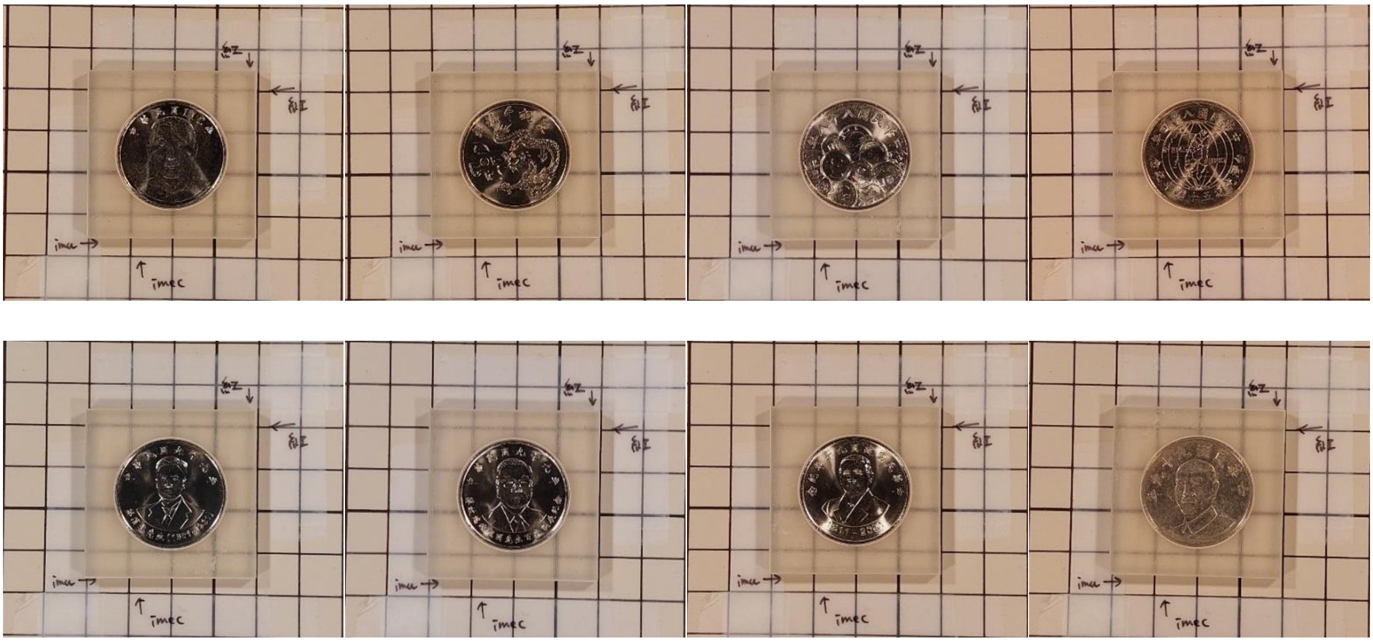

(e)

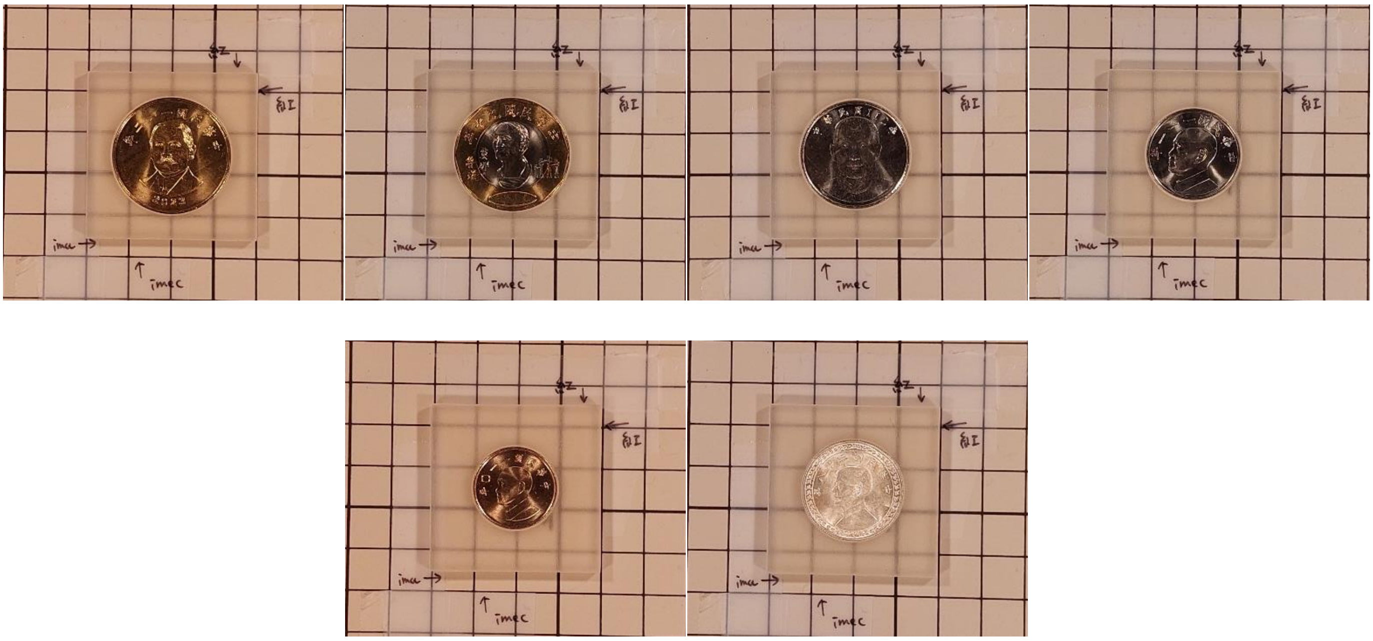

(f)

Supplementary Figure S1. Actual coin samples used in the different SWIR-HSI experiments. (a) Twenty-five independent new and uncirculated NT$10 coins issued in ROC Year 112 and used for imaging-position and aperture optimization; (b) NT$10 coin used for the support-material comparison; (c) the same NT$10 coin used for the rotation-angle experiment, shown at in-plane orientations of 0°, 90°, 180°, and 270° from left to right; (d) new and circulated NT$10 coins used for the surface-use-condition comparison; (e) eight independent NT$10 coins representing different design variants; and (f) six independent coins representing different denominations and a special-year coin. The photographs are provided to document the actual physical samples used in each experiment.

Supplementary Table S1. Detailed information on the independent coin samples used in the six primary experiments

Experiment	Denomination / sample category	No. of independent coins	Year / issue information	Surface-use condition	Source / selection
Imaging-position and aperture optimization	NT$10 coins	25	ROC Year 112	New, uncirculated	Randomly selected from a batch of 200 new, uncirculated NT$10 coins obtained from the Bank of Taiwan
Support-material comparison	NT$10 coin	1	ROC Year 112	New, uncirculated	One coin randomly selected from the same batch of 200 new, uncirculated NT$10 coins
Rotation-angle comparison	NT$10 coin	1	ROC Year 112	New, uncirculated	One coin randomly selected from the same batch of 200 new, uncirculated NT$10 coins
New-versus-circulated comparison	NT$10 coins	2<br>(1 new + 1 circulated)	New coin: ROC Year 112<br>Circulated coin: ROC Year 111	One new, uncirculated coin and one circulated coin	New coin: randomly selected from the same batch of 200 new coins<br>Circulated coin: randomly selected from coins available in the laboratory
Design-variant comparison	Eight NT$10 design variants	8<br>(1 per design variant)	Various design variants; issue year was not systematically used as a grouping variable for all variants	Representative coins; no predefined standardized wear-level grouping	One representative independent coin for each design variant; actual samples are documented in Supplementary Figure S1
Denomination comparison	NT$50, NT$20, NT$10, NT$5, NT$1, and NT$0.5	6<br>(1 per category)	NT$0.5: ROC Year 38<br>Other denominations: issue year was not used as a grouping variable	Representative coins; no predefined standardized wear-level grouping	One representative independent coin for each denomination or special-year category; actual samples are documented in Supplementary Figure S1

Notes: ROC = Republic of China. "Independent coins" refers to physically distinct coin samples within each experiment. The source batch comprised 200 new, uncirculated NT$10 coins issued in ROC Year 112 and obtained from the Bank of Taiwan. For the design-variant and denomination comparisons, one representative coin was used per category; these comparisons were intended for controlled spectral comparison rather than population-level inference.

Supplementary Table S2. Complete mean spectral standard deviations at 25 positions under different coin sides and aperture conditions Supplementary Table S2 lists the complete mean spectral standard deviations at 25 positions under six imaging conditions: F1.8_H, F2.8_H, F4_H, F1.8_W, F2.8_W, and F4_W.

Aperture \ Positions	1	2	3	4	5	6	7	8	9	10	11	12	13	14	15	16	17	18	19	20	21	22	23	24	25
F1.8_H	0.666	0.214	0.224	0.216	0.570	0.353	0.310	0.169	0.134	0.368	0.277	0.344	0.225	0.247	0.224	0.389	0.206	0.238	0.185	0.492	0.699	0.217	0.232	0.214	0.643
F2.8_H	0.760	0.223	0.370	0.234	0.576	0.368	0.237	0.269	0.162	0.426	0.292	0.233	0.163	0.278	0.234	0.441	0.202	0.350	0.196	0.552	0.718	0.277	0.216	0.247	0.679
F4_H	0.922	0.230	0.508	0.194	0.816	0.509	0.391	0.406	0.287	0.432	0.439	0.412	0.361	0.185	0.290	0.526	0.223	0.445	0.199	0.634	1.162	0.243	0.337	0.191	0.890
F1.8_W	0.740	0.753	0.643	0.696	0.650	0.325	0.199	0.226	0.292	0.245	0.237	0.244	0.192	0.288	0.250	0.369	0.197	0.268	0.275	0.352	1.056	0.960	0.817	0.820	0.983
F2.8_W	0.835	0.904	0.813	0.849	0.597	0.290	0.245	0.224	0.346	0.262	0.323	0.289	0.142	0.282	0.236	0.403	0.219	0.345	0.218	0.314	1.117	1.059	1.053	1.068	1.101
F4_W	1.135	1.102	0.896	0.951	0.622	0.307	0.251	0.235	0.414	0.279	0.354	0.332	0.151	0.332	0.249	0.455	0.220	0.369	0.231	0.329	1.492	1.154	1.040	1.108	1.342

Supplementary Table S3. Stability ranking of 25 positions under different coin sides and aperture conditions Supplementary Table S3 lists the ranking results of the 25 positions under the six imaging conditions, sorted from the lowest to the highest mean spectral standard deviation. A higher ranking indicates lower spectral variation and better imaging stability at that position.

Aperture \ Ranking	1	2	3	4	5	6	7	8	9	10	11	12	13	14	15	16	17	18	19	20	21	22	23	24	25
F1.8_H	9	8	19	17	24	2	4	22	15	3	13	23	18	14	11	7	12	6	10	16	20	5	25	1	21
F2.8_H	9	13	19	17	23	2	12	15	4	7	24	8	22	14	11	18	6	3	10	16	20	5	25	21	1
F4_H	14	24	4	19	17	2	22	9	15	23	13	7	8	12	10	11	18	3	6	16	20	5	25	1	21
F1.8_W	13	17	7	8	11	12	10	15	18	19	14	9	6	20	16	3	5	4	1	2	23	24	22	25	21
F2.8_W	13	19	17	8	15	7	10	14	12	6	20	11	18	9	16	5	3	1	4	2	23	22	24	25	21
F4_W	13	17	19	8	15	7	10	6	20	14	12	11	18	9	16	5	3	4	23	2	24	1	22	25	21
